# TYK2-induced phosphorylation of Y640 suppresses STAT3 transcriptional activity

**DOI:** 10.1038/s41598-017-15912-6

**Published:** 2017-11-21

**Authors:** Raffaele Mori, Joris Wauman, Laura Icardi, José Van der Heyden, Lode De Cauwer, Frank Peelman, Karolien De Bosscher, Jan Tavernier

**Affiliations:** 1 Receptor Research Laboratories, Cytokine Receptor Lab, VIB-UGent Center for Medical Biotechnology, 9000, Ghent, Belgium; 2 Receptor Research Laboratories, Nuclear Receptor Lab, VIB-UGent Center for Medical Biotechnology, 9000, Ghent, Belgium; 3 Department of Biochemistry, Ghent University, Ghent, Belgium; 4 Present Address: Università vita-salute San Raffaele, Via Olgettina Milano, 58, 20132 Milano, Italy; 5 Present Address: Argenx BVBA Industriepark Zwijnaarde 7, 9052 Zwijnaarde, Ghent, Belgium; 6 Cancer Research Institute Ghent (CRIG), Ghent, Belgium

## Abstract

STAT3 is a pleiotropic transcription factor involved in homeostatic and host defense processes in the human body. It is activated by numerous cytokines and growth factors and generates a series of cellular effects. Of the STAT-mediated signal transduction pathways, STAT3 transcriptional control is best understood. Jak kinase dependent activation of STAT3 relies on Y705 phosphorylation triggering a conformational switch that is stabilized by intermolecular interactions between SH2 domains and the pY705 motif. We here show that a second tyrosine phosphorylation within the SH2 domain at position Y640, induced by Tyk2, negatively controls STAT3 activity. The Y640F mutation leads to stabilization of activated STAT3 homodimers, accelerated nuclear translocation and superior transcriptional activity following IL-6 and LIF stimulation. Moreover, it unlocks type I IFN-dependent STAT3 signalling in cells that are normally refractory to STAT3 transcriptional activation.

## Introduction

The signal transducers and activators of transcription (STATs) are transcription factors able to carry specific signals from the cell membrane to the nucleus and induce gene transcription. The STAT protein family comprises seven different members (STAT1, STAT2, STAT3, STAT4, STAT5a, STAT5b, STAT6) that are encoded by distinct genes^1^. All STATs are characterized by six conserved domains: an N-terminal domain (NTD), a coiled-coil domain (CCD), a DNA-binding domain, a linker domain (LD), a Src homology 2 domain (SH2) and a C-terminal transactivation domain (TAD). Different ligands can activate STAT proteins, including growth factors, hormones and cytokines. Also mutated kinases, observed in neoplastic disease, are able to activate STAT proteins^2–4^. Abnormal STAT signalling may lead to chronic inflammatory diseases and cancer development^5,6^. Therefore the role of STATs in numerous physiological and pathological processes including cell survival, immunity, angiogenesis, metastasis and oncogenesis is being investigated in great detail^7–11^. According to the canonical model, STATs are activated by cell-surface receptors associated with tyrosine kinases of the JAK (Janus Tyrosine Kinase) family. JAK members include JAK1, JAK2, JAK3 and TYK2^7^. Following structural analyses of JAKs, four distinct domains are identified: an N-terminal FERM domain and an SH2-like domain responsible for binding to the intracellular region of the cognate receptor^12^, followed by a central kinase-like (KL) domain and a C-terminal tyrosine kinase (TK) domain. The KL domain harbors an auto- and trans-regulatory activity that either keeps the protein in an inactive or low phosphorylated state in absence of ligand^13^ or stabilizes the enzyme in an activated state upon cytokine stimulation^14^. Upon ligand-binding to the receptor, JAKs are activated by trans-phosphorylation of key tyrosines in the activation loop of the TK domain^15^ and in turn phosphorylate specific tyrosine residues on the intracellular domains of the associated receptor chain. This process creates docking sites for the recruitment of proteins containing a Src homology 2 (SH2) domain, such as STATs. Upon recruitment to the receptor, STATs are phosphorylated and can form activated parallel homo- and hetero-dimers stabilized by reciprocal, intermolecular interactions between the phosphotyrosine of one STAT and the SH2 domain of another STAT. These homo- and hetero-dimers can subsequently translocate to the nucleus and initiate transcription by binding specific sites in the promoters of target genes. In non-canonical models, some features differ from the above outlined mechanism. For instance, also non-phosphorylated STAT dimers are reported to translocate to the nucleus and contribute to gene regulation^16,17^. STATs may control gene expression not only as dimers but also as tetramers at particular gene promoters^18^ or even as conglomerates of a higher molecular mass, called “statosomes”^19^. STAT3 is known to be abnormally activated in autoimmune diseases, chronic inflammation and in a large percentage of human cancers. This is mostly the consequence of aberrant tyrosine phosphorylation by oncogenic receptor tyrosine kinases, JAKs and Src family kinases^20,21^ rather than mutations in STAT3 itself. Similar to the other STAT members, STAT3 activity is tightly controlled by posttranslational modifications including phosphorylation at Y705^22^ and S727^23^, lysine acetylation^24–26^, methylation^27,28^, ubiquitination^29^ and SUMOylation^30^. A plethora of studies identify Y640 as a fundamental residue modulating STAT3 transcriptional activity in response to known STAT3 transcriptional activators like interleukin-6 (IL-6) and leukemia inhibitory factor (LIF) and even to type I IFNs^31–35^. Yet, its role and mechanism of action has remained elusive. Here we show that phosphorylation of Y640 is involved in the negative regulation of STAT3 transcriptional activity in response to different cytokines. Remarkably, only the kinase domain of TYK2 and not of the other JAK kinases is able to catalyze this alternative phosphorylation.

## Results

### The TK domain of TYK2 induces STAT3 dual tyrosine phosphorylation

**Figure 1:**
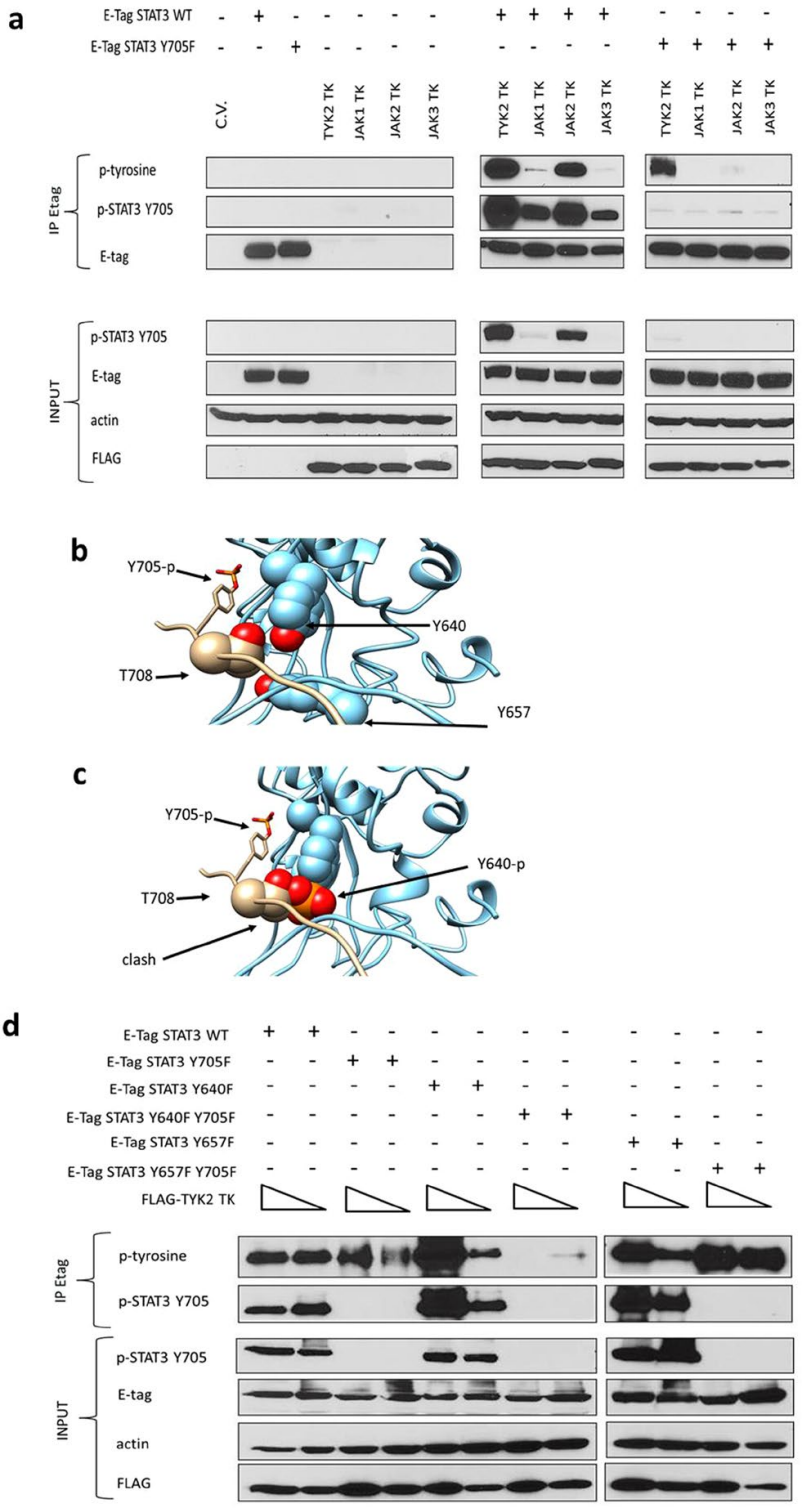
The TYK2 TK domain specifically mediates alternative STAT3 tyrosine phosphorylation. (**a**) HEK293T cells were transiently co-transfected with control plasmid (C.V.) or plasmids coding for Etagged-STAT3 WT or Y705F and FLAG-tagged JAK TK domains. STAT3 was immunoprecipitated with an anti-Etag antibody and the membrane was probed using general anti-phospho-tyrosine, anti-phospho-STAT3 (pY705) and anti-Etag antibodies (panels denoted IP). Total cell lysates were collected before immunoprecipitation, similar amounts of protein extracts were blotted for each condition, as quantified using a Bradford (Bio-Rad) assay and the membrane was probed with anti-phospho-STAT3 (pY705), anti-Etag, anti-β-actin and anti-FLAG antibodies (panels denoted INPUT). n = 2, representative figures. Full-length blots are presented in Supplementary Figs S6–S7. Y640 phosphorylation affects STAT3 dimer formation. (**b**) SH2 domain of STAT3 (blue) with a bound phosphopeptide (pY705) of an interacting STAT3 molecule (beige). T708 and the phosphorylated Y705 of the phosphopeptide are indicated. The hydroxyl group of Y640 and Y657 (red) are in close proximity of the binding site for this phosphopeptide. (**c**) Phosphorylation of Y640 leads to a clash Model of the SH2 domain with a phosphorylated Y640. A STAT3 phosphopeptide was placed in the SH2 domain, as in panel A. This leads to clashes between T708 and the phosphate group of Y640, indicating that Y640 phosphorylation is likely to affect the binding mode with the pY705 phosphopeptide. (**d**) Alternative phosphorylation involving STAT3 Y640. HEK293T cells were transfected with increasing amounts of plasmids coding for FLAG-tagged TYK2 TK domain and different Etagged-STAT3 wt or mutants: STAT3Y705F; STAT3 Y640F; STAT3 Y640F Y705F; STAT3 Y657F; STAT3 Y657F Y705F. STAT3 was immunoprecipitated with an anti-Etag antibody and the membrane was probed using anti-phospho-tyrosine and anti-phospho-STAT3 (pY705) antibodies (panels denoted IP). For the input, total cell lysates were collected before immunoprecipitation and similar amounts of protein extracts were blotted for each condition, as quantified using a Bradford assay and the membrane was probed with anti-phospho-STAT3 (pY705), anti-Etag, anti-β-actin and anti-FLAG antibodies (panels denoted INPUT). n = 2, representative figures. Full-length blots are presented in Supplementary Figs S8–S9.

The STAT3 sequence is extremely conserved, including all the tyrosine residues. Analysis of the available STAT3 crystal structures learns that most tyrosines are at least partially solvent-exposed at the protein surface and could be targeted by posttranslational modifications such as phosphorylation. For instance, Y705 is known to be phosphorylated upon receptor triggering and pY705 is required for formation of parallel activated STAT3 homodimers, nuclear translocation and STAT3-dependent gene transcription^36–39^. To test whether additional tyrosines could be targeted by the JAKs, we used an unbiased approach by transiently co-expressing constitutively active tyrosine kinase domains (FLAG-TKs) of JAK1, JAK2, JAK3 or TYK2 together with either wild type (wt) or Y705F mutant E-tagged-STAT3 in HEK293T cells. Immunoprecipitation analysis demonstrated that, as expected, all four JAK TKs can induce Y705 phosphorylation of wt STAT3 (Fig. 1a) with stronger signals for TYK2 and JAK2 compared to JAK1 and JAK3. Remarkably, only inclusion of TYK2 TK revealed the existence of an additional tyrosine phosphorylation site in STAT3 Y705F when probing with a monoclonal anti-pY antibody (Fig. 1a).

PDBe PISA^40^ analysis of the STAT3 crystal structure^41^ indicates that two tyrosines of the SH2 domain are buried in the STAT3 dimerization interface (Fig. 1b). Y640 of one STAT3 is present in the binding cleft of the peptide sequence immediately downstream of Y705 of the other STAT3 and 23 Å^2^ of its surface becomes buried below T708 upon STAT3 dimerization. Y657 is present close to this binding cleft, but this residue is less solvent exposed and only 0.29 Å^2^ of its surface becomes buried after STAT3 dimer formation. Molecular modelling indicates that the phosphorylation of Y640 is likely to interfere with STAT3 dimerization (Fig. 1c): the phosphate introduced at Y640 partially occupies the binding site for T708, a threonine that was already shown to be important for STAT3-DNA complex formation^42^. Given the crucial role for the pY705-SH2 domain interaction^43^ we next performed a similar IP analysis as described above using wt or Y705F Etag-STAT3 carrying the additional Y640F or Y657F mutations. As shown in Fig. 1d, immunoblotting using the anti-pY antibody showed complete loss of the TYK2 TK-induced phosphorylation signal, only in case of the double Y640F-Y705F mutant. Taken together, these data provide evidence for a TYK2-driven phosphorylation of Y640, in addition to phosphorylation of the canonical Y705 motif.

### Mutation of Y640 enhances formation and stability of activated STAT3 homodimers in response to IFNα2

**Figure 2:**
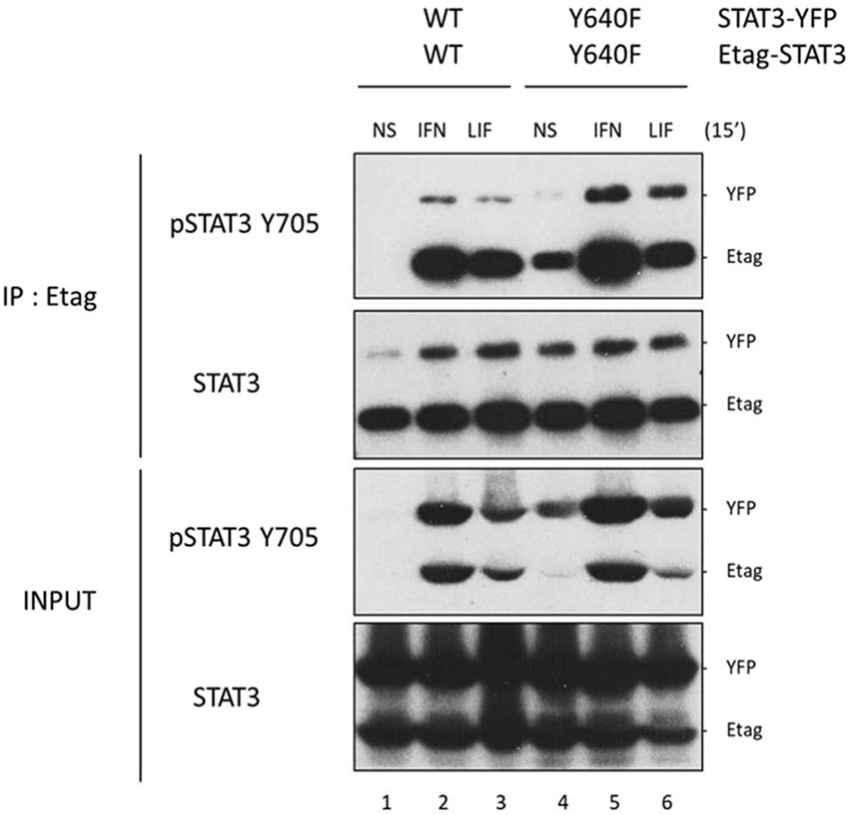
Enhanced IFNα2-induced homodimerization of the STAT3 Y640F mutant. HEK293T cells were transiently transfected either with the combination of Etag-STAT3wt and STAT3wt-YFP or with the combination of Etag-STAT3 Y640F and STAT3 Y640F-YFP. Cells were serum starved 6 hours and then stimulated for 15 minutes with 5 ng/ml IFNα2 or 20 ng/ml LIF, or left untreated. Etag-STAT3 was immunoprecipitated with an anti-Etag antibody. Total STAT3 and phosphorylated STAT3 were revealed with an anti-STAT3 and anti-STAT3 pY705 antibodies, respectively (panels denoted IP). Total cell lysates were collected before immunoprecipitation, protein concentration was determined by Bradford assay and similar amounts of protein extracts were blotted for each condition, using antibodies as indicated in the Figure (panels denoted INPUT). n = 3, representative figures. Full-length blots are presented in Supplementary Figs S10–S11.

We next analysed whether the Y640F mutation may affect the ligand-induced homodimerization status of STAT3. E-tagged and YFP-tagged versions of either STAT3 wt or STAT3 Y640F were transiently co-expressed in HEK293T cells. Cells were treated with IFNα2 or LIF for 15 minutes and Etag-STAT3 (either wt or mutant) was immunoprecipitated. Western blot analysis was used to reveal STAT3 phosphorylation and dimerization. As expected, both IFNα2 and LIF stimulation induced phosphorylation and dimerization of wild-type STAT3 and this was further enhanced when combining the STAT3 Y640F mutants (Fig. 2). The effect is however more pronounced for IFNα2, which is in line with TYK2 being a key JAK kinase of the IFN receptor (IFNAR) complex (Fig. 2, upper panel, lane 5 versus lane 2). Interestingly, the input control demonstrates enhanced phosphorylation of Y705-STAT3 in the untreated condition, only when STAT3 is mutated at Y640 (Fig. 2, lower panels, lane 4). This observation is completely in line with earlier findings by Pilati *et al*.^31^ that in the presence of STAT3 Y640F a significant fraction of STAT3 is constitutively phosphorylated and dimerized. To conclude, STAT3 Y640F leads to enhanced phosphorylation of STAT3 at Y705 and to increased dimerization particularly after IFNα2 stimulation.

### STAT3 Y640F shows increased nuclear translocation and binding at the *SOCS3* promoter

**Figure 3:**
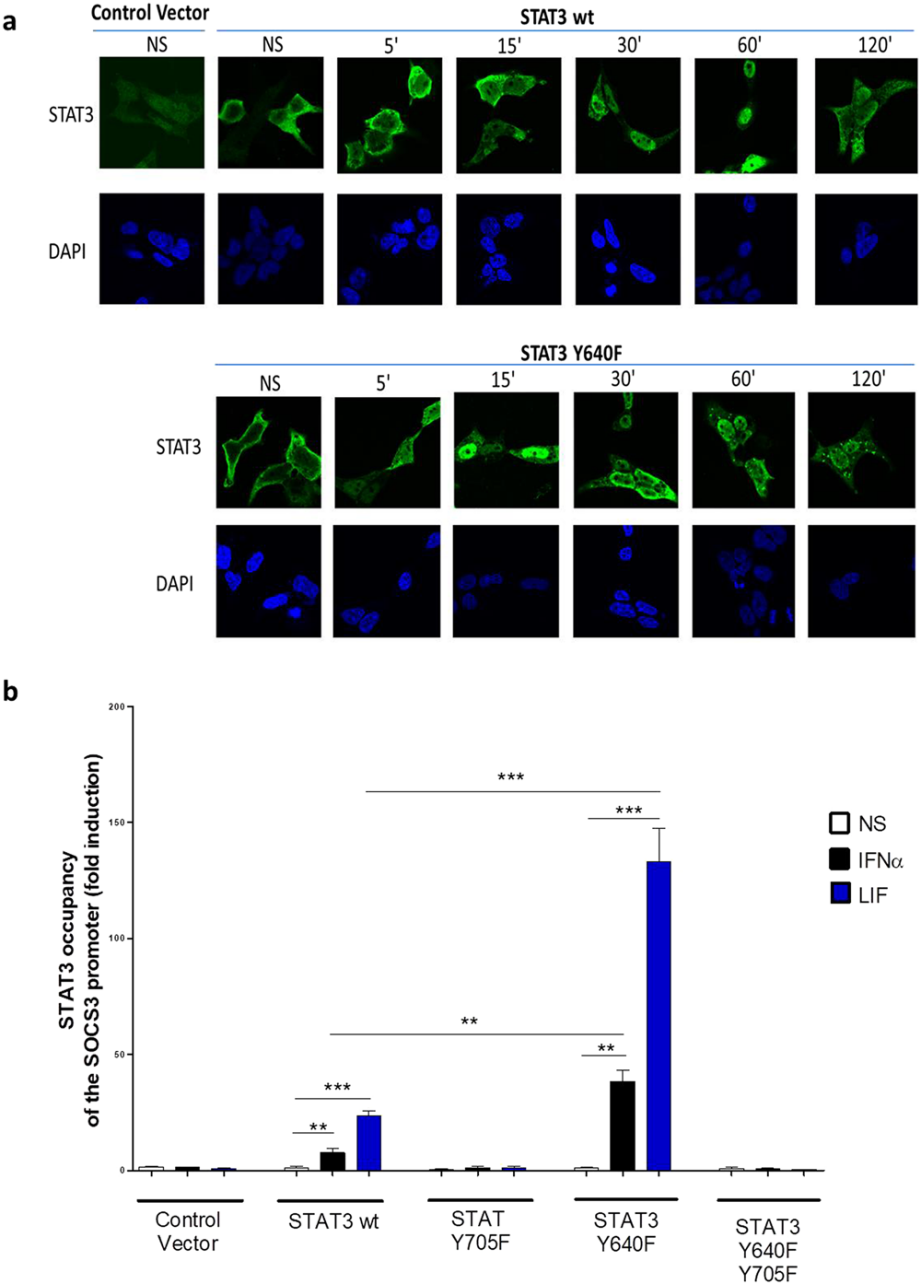
The STAT3 Y640F mutation influences STAT3 subcellular distribution in response to IFNα2. (**a**) HEK293T cells were transiently transfected with empty vector (Control Vector), Etag-STAT3 wt or Etag-STAT3 Y640F mutant. Cells were starved for 16 hours and then either left unstimulated (NS) or stimulated with IFNα2 (10ng/ml) for the indicated times. Cells were fixed, stained with DAPI and anti-Etag and the localization of STAT3 was assessed by confocal analysis. Immunofluorescence of representative cell fields is shown. Enhanced IFNα2-induced DNA binding of the STAT3 Y640F mutant. (**b**) ChIP assays were performed to examine the occupancy of STAT3 on the SOCS3 promoter. HEK293T cells were transiently transfected either with empty vector (Control Vector) or different Etag-STAT3 mutants: STAT3Y705F; STAT3 Y640F; STAT3 Y640F/Y705F. Cells were serum starved 4 hours and then left unstimulated (NS) or stimulated for 1 hour either with 10ng/ml IFNα2 or LIF. Immunoprecipitated DNA was used for qRT-PCR using specific primers for the SOCS3 promoter. Graphs represent occupancy levels relative to irrelevant IgG immunoprecipitated DNA samples. All results are representative of 3 independent experiments. Error bars indicate SD. **P < 0.01, ***P < 0.001; 1-way ANOVA with Bonferroni test.

We next analyzed the effect of the Y640F mutation on STAT3 nuclear translocation and DNA binding using immunofluorescence and chromatin immunoprecipitation (ChIP), respectively. We focused on IFNα2, given the pronounced promoting effects of STAT3 Y640F on formation of activated homodimers as observed above. We found that the IFNα2-induced nuclear accumulation of STAT3 was significantly faster in HEK293T cells transiently transfected with Etag-STAT3 Y640F compared to Etag-STAT3 wt expressing cells. The latter cells showed a clear nuclear accumulation only upon 15 min of stimulation with IFNα2 (Fig. 3a). Of note, at later time points (1h-2h) we observed distinct nuclear and perinuclear STAT3 speckles only in STAT3 Y640F mutant transfected cells. As expected, no nuclear translocation was observed for the STAT3 dominant negative Y705F mutant (data not shown).

ChIP assays were performed on HEK293T cells transiently expressing Etag-STAT3 wt or the Etag-STAT3 Y640F, Y705F, Y640F/Y705F mutants. Cells were stimulated with either IFNα2 or LIF and as a representative model for STAT3-binding we studied recruitment to the endogenous *SOCS3* promoter. As shown in Fig. 3b, both treatments induced a much more pronounced recruitment of STAT3 Y640F compared to STAT3 wt. The Y705F mutant and the dual mutant Y640F/Y705F showed no signs of S*OCS3* promoter binding. For all the condition tested there was no evidence for binding at the endogenous promoter of the non STAT3-responsive *HPRT* gene (Suppl. Data Fig. S1). In conclusion, our data show that the Y640F mutation speeds up the nuclear translocation of STAT3 dimers following IFNα2 treatment, facilitating an enhanced binding to the STAT3-responsive gene *SOCS3*. EMSA experiments confirmed the results obtained by ChIP analysis using *SOCS3* and *cFOS* promoter sequences as probes. In concordance with the ChIP experiments, the STAT3 Y640F mutant showed enhanced binding to the probes as compared to STAT3 wt upon both IFNα2 and LIF stimulation (Suppl. Data Fig. S2).

### Expression of the STAT3 Y640F mutant restores IFNα2-dependent STAT3 transcriptional activity

**Figure 4:**
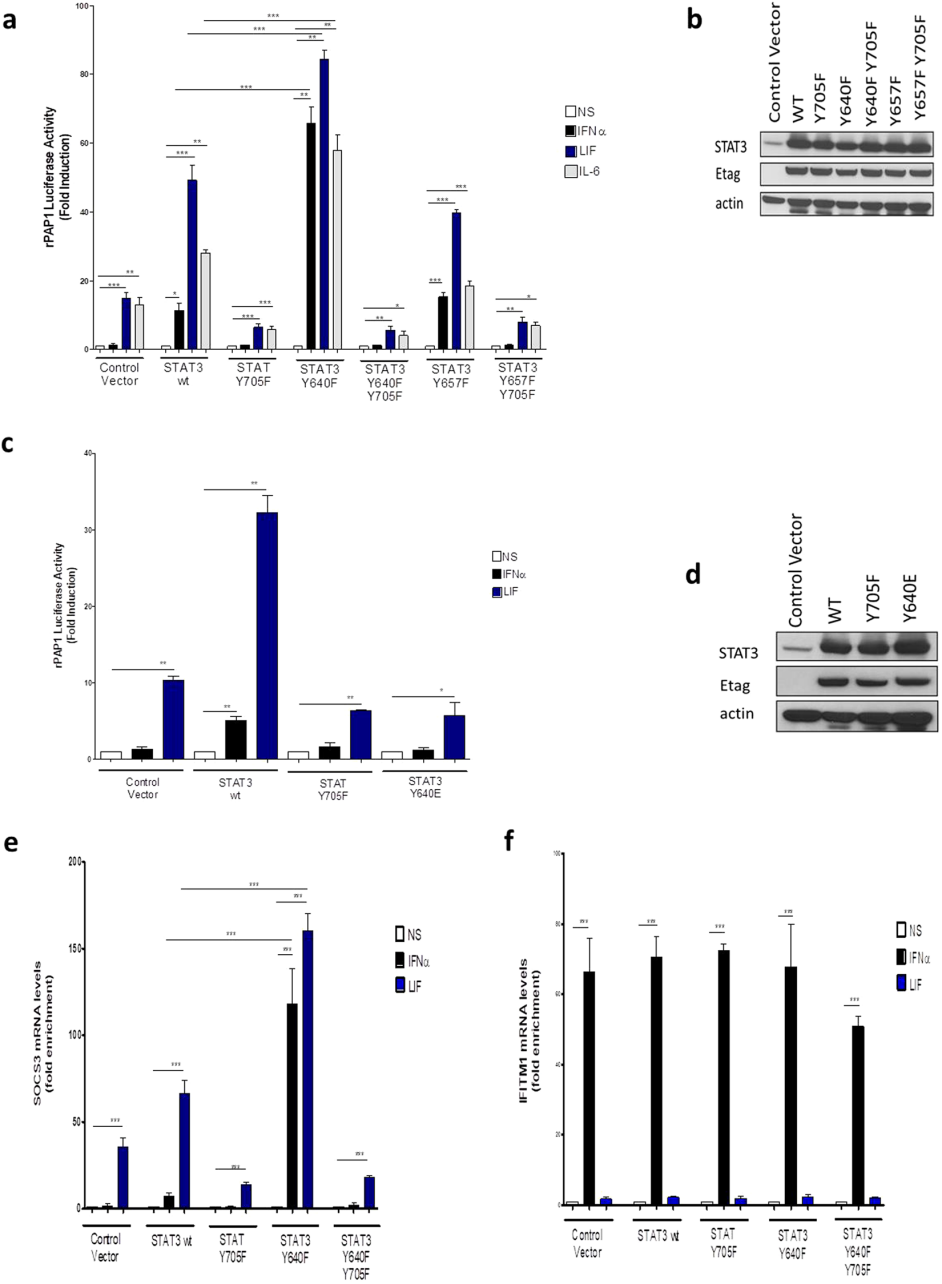
Mutation of STAT3 at Y640 restores IFNα2-induced STAT3 transcriptional activity. (**a**) Left panel: HEK293T cells were transiently transfected with the pXP2d2-rPAP1-luciferase reporter together with the plasmid coding for the IL-6Rα chain and the control plasmid (Control Vector) or plasmids coding for different Etag-STAT3 mutants: STAT3Y705F; STAT3 Y640F; STAT3 Y640F Y705F; STAT3 Y657F; STAT3 Y657F Y705F. Cells were left unstimulated (NS) or stimulated for 24 hours with 10ng/ml IFNα2 or LIF or with 100ng/ml IL-6. Luciferase readout is expressed as a ratio between stimulated and unstimulated values. All results are representative of 3 independent experiments. Error bars indicate SD. *P < 0.05, **P < 0.01, ***P < 0.001; Student t-test. (**b**) Right panel: total cell extracts were blotted and membranes were probed with anti-STAT3, anti-Etag and anti-β-actin antibodies. Full-length blots are presented in Supplementary Fig. S12. (**c**) HEK293T cells were transiently transfected with the pXP2d2-rPAP1-luciferase reporter together with the control plasmid (Control Vector) or plasmids coding for different Etag-STAT3 mutants: STAT3Y705F; STAT3 Y640E. Cells were left unstimulated (NS) or stimulated for 24 hours with 10ng/ml IFNα2 or LIF. Luciferase readout is expressed as a ratio between stimulated and unstimulated values. All results are representative of 2 independent experiments. Error bars indicate SD. *P < 0.05, **P < 0.01; Student t-test. (**d**) Total cells extracts were blotted to test transfection efficiencies and the membranes probed with anti-STAT3, anti-Etag and anti-β-actin antibodies. Full-length blots are presented in Supplementary Fig. S13. qRT-PCR analysis representing the relative mRNA levels of genes (**e**) SOCS3 and (**f**) IFITM1. HEK293T cells were transiently transfected with empty vector (control vector) or different Etag-STAT3 mutants: STAT3Y705F; STAT3 Y640F; STAT3 Y640F Y705F. Cells were serum-starved 4 hours and then left unstimulated (NS) or stimulated for 24 hours either with 10ng/ml IFNα2 or LIF. Graphs represent the mRNA levels relative to the non-stimulated samples. All results are representative of 3 independent experiments. Error bars indicate SD. ***P < 0.001; 1-way ANOVA with Bonferroni test.

STAT3-driven gene transcription has been shown to be tightly controlled by cell-specific events, including post-translational modifications. We previously reported that in HEK293T cells, transcriptional activity of STAT3 was undetectable upon stimulation with IFNα2^44^, in marked contrast to LIF, although Y705 phosphorylation was comparable for both stimuli. In this context and given the abovementioned critical role of TYK2, we wondered whether phosphorylation of STAT3 Y640 could serve as an inhibitory phosphorylation event hampering STAT3-dependent gene expression upon type I IFN signalling. We first evaluated the well-documented STAT3-responsive rPAP1-luciferase reporter^45^. HEK293T cells were transiently co-transfected with the reporter vector, a plasmid coding for the IL-6Rα chain and one of the following Etag-STAT3 constructs: wt, Y705F, Y640F, Y640F/Y705F, Y657F or Y657F/Y705F, followed by stimulation with IFNα2, LIF or IL-6. Interestingly, we found that the presence of the Y640F mutant strongly increased reporter activity for all cytokines tested, but the effect was far most pronounced for IFNα2, with an induction comparable to the other cytokines (Fig. 4a,b).

Further experiments also showed that expression of Y640F mutant in murine N38 and NIH3T3 cells, transiently co-transfected with the STAT3-responsive rPAP1-luciferase reporter, leads to a pronounced increase of STAT3 transcriptional activity upon IFNα or LIF treatment compared to cells expressing STAT3 wt thus providing evidence that cell lines other than HEK293T have a similar regulatory mechanism involving STAT3 phosphorylation at Y640 (Suppl. Data Figs S3–S4).

This upregulation was specific for the Y640F mutation since no transcriptional enhancement was observed for the Y657F mutant. Expression of STAT3 wt led to moderately increased SOCS3 expression upon LIF stimulation and to some extent also after IFNα2 stimulation, in accordance with previous studies showing restoration of IFNα2-dependent STAT3 transcriptional activity in HEK293T cells overexpressing STAT3 (data not shown). We further evaluated the effect of the phospho-mimicking STAT3 Y640E mutant on activation of the rPAP1 reporter (Fig. 4c,d). HEK293T cells expressing the Y640E mutant showed strongly impaired LIF-dependent reporter transcription, comparable to the Y705F mutant, further illustrating the dominant negative activity of this mutant on activated endogenous STAT3.

Next, we also measured transcriptional activity by qPCR for the endogenous STAT3-responsive *SOCS3* gene in HEK293T cells transiently transfected with STAT3 wt or mutants and stimulated with either IFNα2 or LIF. Importantly, a dramatic increase was again observed when expressing the STAT3 Y640F mutant, whereby IFNα2-dependent transcription almost equalled the increased LIF-induced transcription (Fig. 4e). In the same experiment, expression of Y705F and Y640F-Y705F mutants led to reduced LIF-induced *SOCS3* transcription. Type I IFNs also activates another STAT dependent signalling cascade relying on STAT1, STAT2 and IRF9 which form the ISGF3 complex^46^. Transcription of the ISGF3-responsive genes *IFITM1* (Fig. 4f) and 2′5′*OAS* (Suppl. Data Fig. S5) was unaffected by wt or mutant STAT3 expression. Taken together, our results show that phosphorylation of Y640 is an inhibitory posttranslational modification. In accord, mutation hereof can enhance STAT3-dependent transcription, and, in case of type I IFN signalling, can even override a complete transcriptional block.

## Discussion

The precise role of STAT3 in type I IFN signaling has been a matter of debate due to the complex nature of STAT3 itself and to its diverse functions that strongly depend on the cellular context^47–53^. STAT3 has been shown to mostly promote proliferation, cell cycle progression and survival^54^ whilst type I IFN-driven transcription involves genes with often pro-apoptotic activities^55^, indicating that mechanisms must exist to fine tune the transcriptional activity of STAT3 in a cytokine- and cell-dependent way. We previously reported that the transcriptional activity of IFNα2-activated STAT3 can be regulated at the nuclear level by the Sin3a co-repressor complex that exerts an inhibitory activity downstream of STAT3-DNA binding^44^. We showed that IFNα2 stimulation leads to STAT3 phosphorylation, nuclear translocation and DNA-binding comparable as observed upon LIF treatment but without inducing a comparable transcriptional activity. Sin3a was found to function as a scaffold for other proteins in the complex by directly interacting with acetylated STAT3 thereby promoting STAT3 deacetylation and inhibiting STAT3-driven gene transcription^56^. Acetylation together with phosphorylation and other post-translational modifications (PTMs) have been reported to influence STAT3 signaling, possibly involving cross-talk mechanisms, that ultimately govern the complex modulation of STAT3 activity^57,58^. Our findings now point to a surprising new role for TYK2 as a kinase that can impair STAT3 transcriptional activity. Enhanced STAT3 reporter activation and gene transcription are observed for IL-6 and LIF, which signal through gp130-based receptor complexes, but the enhanced transcriptional effect upon mitigation of the TYK2-targeted phosphorylation site is most pronounced in response to IFNα2. This is not unexpected since TYK2 is the key kinase associated to the IFNAR1 chain of the IFNAR complex. This is in contrast to signaling through gp130-based cytokine receptors complexes, which rely mainly on JAK1-JAK2 activity, where TYK2 functions more like a bystander kinase^59–64^.

In line with these findings, only the TK domain of TYK2 and not of any other JAK was capable of inducing an alternative phosphorylation in STAT3 Y705F mutant co-expressing cells. This led us to hypothesize the presence of an alternative STAT3 phosphorylation site which, based on structural considerations involved in engaging the active STAT3 dimer, was identified as the Y640 residue. Its critical role in down-modulating STAT3 transcriptional activity was subsequently demonstrated using dimerization, nuclear translocation, ChIP, reporter and qPCR assays using the Y640F STAT3 mutant. In line with a receptor-proximal control mechanism, we could not detect any variability on histone acetylation or any variation of co-factor recruitment on the *SOCS3* promoter, as a consequence of the Y640F mutation (data not shown). Of note, phospho-specific antibodies directed against pY640 cross-react with the pY705 site and vice versa, since both are embedded in a highly similar sequence motif. Y640 phosphorylation can thus only be detected using a Y705F mutant and not using wt STAT3, possibly explaining why this regulatory phosphosite has remained undetected so far. Notwithstanding, the STAT3 Y640F mutation was already described as one of the most common STAT3 somatic mutations associated with the onset of several cancers, both hematological and solid^31,32^. Y640F mutated STAT3 was found to be constitutively phosphorylated at tyrosine 705 and to dimerize in absence of any stimulus. Moreover, STAT3 Y640F showed a hyper-responsiveness to IL-6 treatment, as compare to STAT3 wt, with an increased transcription of its downstream target genes. IL-6 dependent phosphorylation at S727 was not increased in STAT3 Y640F when compared to IL-6 activated STAT3 wt^31^. These results show that Y640 is a key residue in modulating STAT3 activity and thus can be target of a negative regulatory phosphorylation event. In this study, we demonstrate that STAT3 Y640F is able to respond to IFNα2 showing accelerated nuclear translocation and increased DNA-binding, as compared to IFNα2-activated STAT3 wt, ultimately resulting in a remarkable IFN-dependent transcriptional activity. In line with our findings, it was recently reported that in anaplastic large cell lymphomas (ALCLs) constitutively active Tyk2 fusion proteins are oncogenic by driving the STAT3 signaling pathway^65^.

Tyrosine 640 is located in the inside a PYTK sequence motif conserved in STAT1, STAT2, STAT3 and to a lesser extent in STAT4. Interestingly, in STAT5α, STAT5β and STAT6 the tyrosine residue within this motif is replaced by a phenylalanine residue. The importance of STAT2 Y631 (the equivalent of STAT3 Y640) has been already described showing that mutating this residue to phenylalanine leads to resistance to dephosphorylation by the nuclear tyrosine phosphatase TcPTP and thus to sustained IFN-dependent STAT1 Y701 phosphorylation, STAT1-STAT2 Y631F heterodimerization and induction of ISGF3-responsive genes with consequent enhanced cell apoptosis^66^. It has already been reported that STAT molecules can be found intracellularly as preformed dimers in a latent and unphosphorylated state^67,68^. In the same context, STAT3 was found to have a peculiar behavior in being the only STAT protein with two different parallel orientations: one for the latent dimers and one for the activated dimers, both different from the antiparallel orientation of the latent dimers of STAT1 and STAT5^69^. In STAT3, Y640 can be targeted by a downregulating phosphorylation, impairing the molecular switch needed for the formation of a parallel activated dimer from a parallel latent dimer. Conversely, the Y640F mutation is predicted to result in a more hydrophobic surface^32^ which might lead to an enhanced stability of activated STAT3 dimers.

The activity of IFN-activated STAT3 varies in a cell context-dependent way. Indeed, depending on the cell type, the level of IFN-induced STAT3 phosphorylation is not followed by a comparable activation of STAT3-responsive genes, whilst in others STAT3 can induce ISGs transcription in response to IFNs. This is supported by a number of studies concerning IFN-driven antiviral and antiproliferative effects in IFN-resistant Daudi cells^70^, IFN-dependent pro-apoptotic activity in primary murine pro-B cells^71^ and IFNα-dependent inhibition of viral replication of Influenza and Vaccinia viruses in STAT3-/- Murine Embryonic Fibroblasts (MEFs)^72^. On the basis of these observations one might speculate about the existence of cell-context dependent fine-tuning of IFN-induced STAT3 activity by e.g. a protein tyrosine phosphatase acting on Y640 phosphorylated STAT3 and releasing the IFN-specific transcriptional brake on STAT3. Finally, Src kinases are also known to regulate STAT3 phosphorylation and therefore STAT3 activity. We could however rule out a role for Src kinases since the selective inhibitor PP2 did not lead to an upregulation of either IFN- or LIF-induced STAT3-dependent transcriptional activity in HEK293T cells transiently expressing either STAT3 wt or STAT3 Y640F (data not shown).

In conclusion, we demonstrate that mitigation of the STAT3 Y640 phosphorylation, as demonstrated by the Y640F mutation, renders STAT3 transcriptionally competent in response to IFNα2. We suggest a key role for the tyrosine kinase activity of TYK2 in the repression of STAT3 activity. The proposed mechanism may serve to fine-tune IFN-specific effects in different cell types.

## Materials and Methods

### Plasmids, cytokines and treatments

The STAT3-responsive rat pancreatitis-associated protein 1 (rPAP1) promoter was used to generate pXP2d2-rPAP1-luciferase reporter as described elsewhere^45^. The plasmid pMET7-Etag-STAT3wt was constructed using the primer pair 5′-GCGGCGGCCGCAGCCCAATCAGCTACAG-3′ and 5′-CGCCTCGAGTCACATGGGGGAGGTAGCGCA-3′ to clone STAT3 into pMET7 vector using NotI-XhoI. By site-directed mutagenesis we generated STAT3 mutants starting from pMET7-Etag-STAT3 wt using the mutagenesis primers: Y705F 5′-GGTAGCGCTGCCCCATTCCTAAAG ACCAAGTTTATCTG-3′ and 5′-CAGATAAACTTGGTCTTTAGGAATGGGGCAGCGCTACC-3′; Y640F 5′-GATCCAGTCCGTGGAACCATTCACAAAGCAGCAACTGAACAACATGTCATT T-3′ and 5′-CAAATGACATGTTGTTCAGTTGCTGCTTTGTGAATGGTTCCACGGACTGGAT C- 3′; Y640E 5′-GATCCAGTCCGTGGAACCAGAGACAAAGCAGCAACTGAACAACATGT CATTTG-3′ and 5′-CAAATGACATGTTGTTCAGTTGCTGCTTTGTCTCTGGTTCCACGGAC TGGATC-3′; Y657F 5′-GAAATCATCATGGGCTTTAAAATCATGGATGC- 3′ and 5′-GCATCC ATGATTTTAAAGCCCATGATGATTTC-3′. The TK domains of the four different JAKs were cloned in the pMET7-FLAG vector using EcoRI-NotI after PCR with primers: 5′-GACGGAATTCATGGACCCTACGGTTTTCCAC-3′ and 5′-GACGGCGGCCGCTCAGCACA CG-3′ (TYK2); 5′-GACGGAATTCATGGACCCCACACATTTTGAAAAGC-3′and 5′-GACGGC GGCCGCTTATTTTAAAAG-3′ (JAK1); 5′-GACGGAATTCATGGATCCTACACAGTTTGAAG AG- 3′ and 5′-GCAGGCGGCCGCTCATCCAGCCATG-3′ (JAK2); 5′-GACGGAATTCATGCA AGACCCCACGATCTTCGAGGAG-3′ and 5′-GACGGCGGCCGCCTATGAAAAGGACAG-3′ (JAK3). Recombinant human IFNα2, recombinant mouse IFNαA (PBL Interferon Source, Piscataway, NJ, USA) and recombinant human LIF (Millipore, Billerica, MA, USA) were used at 10 ng/ml, recombinant human IL-6 (Peprotech) was used at 100 ng/ml.

### Cell culture, plasmid transfection and luciferase assay

HEK293T cells were grown in DMEM + 10% FCS and transiently transfected overnight using a standard calcium phosphate precipitation procedure^73^. For immunoprecipitation experiments, 2 × 10^5^ HEK293T cells were seeded in each well of a 6 well plate per condition. The next day cells were transfected with 1.5 µg of total plasmid DNA for each well: 0.2 µg of pMET7-Etag-STAT3, 0.5 µg or 0.1 µg pMET7-FLAG-JAKs TK and 0.7 µg or 1.2 µg empty pMET7 vector. For STAT3 dimerization experiments 1 × 10^6^ HEK293T cells were seeded in a 10 cm dish and transfected the next day with 10 µg of total plasmid DNA: 0.5 µg of pN.1-STAT3-EYFP (WT or Y640F) and 9.5 µg of pMET7-Etag-STAT3 (WT or Y640F). For ChIP experiments, 3 × 10^6^ HEK293T cells were seeded in a 14 cm Petri dish and transfected the next day with 25 µg of total plasmid DNA for each 14-cm dish: 6 µg of pMET7-Etag-STAT3 and 19 µg empty pMET7 vector. For RT-PCR experiments, 2 × 10^5^ HEK293T cells were seeded in each well of a 6 well plate and transfected the next day with 1.5 µg of total plasmid DNA: 0.2 µg of pMET7-Etag-STAT3 constructs and 1.3 µg empty pMET7 vector. For the immunofluorescence analysis 1.5 × 10^5^ Hek293T cells were transfected with 1 µg total DNA in a 6 well plate: 0.2 µg of pMET7-Etag-STAT3 constructs and 0.8 µg empty pMET7 vector. For the luciferase assay in HEK293T cells 1.5 × 10^5^ cells were seeded in each well of a 6 well plate and transfected the next day with 1.5 µg total plasmid DNA: 0.2 µg luciferase reporter vector; 0.2 µg expression vector; 0.1 µg pSVL-hIL-6Ra-HAtag coding IL-6Rα chain; 1 µg empty pMET7 vector. For luciferase assay in murine N38 and NIH3T3 cells 4.5 × 10^5^ cells were seeded in each well of a 6 well plate and transfected the next day using Lipofectamine 2000 Transfection Reagent (Thermo Fisher Scientific) following the manufacturer’s instructions with 2.5 µg total plasmid DNA: 0.2 µg luciferase reporter vector; 0.5 µg expression vector; 1.8 µg empty pMET7 vector. The next day cells were washed with PBS, trypsinized, transferred to a 96-well plate and left untreated or stimulated with the indicated amount of hIFNα2, mIFNαA, hLIF or hIL-6 for 24 h. Luciferase activity was measured by chemiluminescence with a TopCount (Canberra-Packard, Waverley, UK) and expressed as fold induction (stimulated/nonstimulated).

### Lysate preparation, Immunoprecipitation and Western Blot analysis

48 h after transfection, HEK293T cells from a single 6 well plate were collected, pooled in ice-cold PBS and lysed with 1 ml of modified RIPA lysis buffer (200 mM NaCl; 50 mM Tris-HCl, pH 8; 0.05% SDS; 2 mM EDTA; 1% Nonidet P-40; 0.5% sodium deoxycholate; 1 mM Na_3_VO_4_; 1 mM NaF and complete Protease Inhibitor Cocktail; Roche Applied Science, Penzberg, Germany). Cleared lysates were incubated with monoclonal mouse anti-Etag antibody (Phadia) overnight at 4 °C. Immune complexes were precipitated by adding 35 µl (50% slurry) of Protein G Dynabeads (Thermo Fisher Scientific) and rotated for 3 h at 4 °C. Beads were washed 3 times with modified RIPA buffer, resuspended in 30 µl of 5xSDS loading buffer and boiled at 98 °C for 10 min. For STAT3 dimerization experiments, 48 hours after transfection cells were serum-starved for 6 hours and stimulated for 15 minutes with 5 ng/ml IFNα or 15 ng/ml LIF, or left untreated. Cells were lysed using 1 mL of modified RIPA buffer (for STAT3 dimers: lysates were homogenized with a dounce homogenizer), lysate supernatans were collected and Etag-STAT3 was immunoprecipitated using 2 µg mouse anti-Etag (Phadia) and 30 µl (50% slurry) Protein G Dynabeads (Thermo Fisher Scientific). Immunoprecipitated proteins were eluted in 2x Laemli buffer and heated at 95 °C for 5 minutes. Lysates were loaded on a 10% SDS-polyacrylamide gel and transferred to a polyvinylidene difluoride (PVDF) membrane (Millipore). The following antibodies were used: mouse monoclonal anti-phosphotyrosine clone 4G10 (Millipore); anti mouse monoclonal anti-Etag (Phadia); mouse monoclonal anti-STAT3 (Cell Signaling Technology); rabbit polyclonal anti-phosphorylated Y705 STAT3 (Cell Signaling Technology); mouse monoclonal anti-β-actin (Sigma Aldrich); mouse monoclonal anti-FLAG (Sigma Aldrich); either peroxidase AffiniPure goat anti-mouse IgG or peroxidase AffiniPure goat anti-rabbit IgG (Jakson, Jakson ImmunoResearch) was used as secondary antibody. SuperSignal pico Chemiluminescent Substrate (Thermo Fisher Scientific) was used as substrate of peroxidase and targeted proteins on the blot were visualized using CL-XPosure films (Thermo Fisher Scientific).

### Chromatin Immunoprecipitation (ChIP) assays

48 hours after transfection cells were serum-starved for 4 hours and stimulated with IFNα2 or LIF for 1 h, followed by fixation with 1% formaldehyde at room temperature for 10 min. Fixation was quenched by addition of 2 M glycine at the final concentration of 10%. Cells were washed once, collected in with PBS + 0.5 mM EDTA and centrifuged for 5 minutes 1500 rpm 4 °C. Cell pellets were resuspended and sonicated in PBS + 0.5 mM EDTA + 0.5% Nonidet P-40 + complete Protease Inhibitor Cocktail to shear the chromatin DNA to fragments of 500–1000 bp. The supernatant was clarified by centrifugation and 1/10 of the chromatin was saved as input. The remaining chromatin solution was incubated with mouse monoclonal anti-Etag (Phadia) or normal mouse Immunoglobulin G (Santa Cruz Biotechnology) at 4 °C overnight. Antibody-protein-DNA complexes were incubated with 40 µl (50% slurry) Protein G Dynabeads (Thermo Fisher Scientific) for 3 h at 4 °C and immunoprecipitates were washed once with RIPA buffer (10 mM Tris-HCl, pH 8; 150 mM NaCl; 0.1% NonidetP-40;0.1% sodium deoxycholate; and Complete Protease Inhibitor Cocktail), twice with RIPA-500 buffer (10 mM Tris-HCl, pH 8; 500 mM NaCl; 0.1% SDS; 1% Nonidet P-40; 0.1% sodium deoxycholate; and Complete Protease Inhibitor Cocktail), once with LiCl buffer (10 mM Tris-HCl, pH8; 250 mM LiCl; 0.5% Nonidet P-40; 0.5% sodium deoxycholate; 1 mM EDTA and Complete Protease Inhibitor Cocktail) and one last time with TBS buffer (20 mM Tris-HCl, pH7.6 and 137 mM NaCl). Immune complexes were eluted from beads using elution buffer (1% SDS; 0.1% NaHCO_3_) at room temperature while shaking. Cross-linking was reverted by incubation at 67 °C overnight. All samples were treated with 50 ng/µl of RNase A and 100 ng/µl of Proteinase K. DNA fragments were purified using the QIAquick PCR purification kit (Qiagen) and subjected to quantitative PCR using SYBR Green Master (Roche Applied Science) with the specific primer pair for the *SOCS3* promoter 5′-AAAAGGGGAAGGGGAACC-3′ and 5′-GGAGAGCGGGCAGTTCTA-3′. The results were analyzed using the ΔΔC_*T*_ method. The fold change of STAT3 promoter occupancy was calculated by normalizing the relative amount to the input and comparing with normal mouse Immunoglobulin G precipitated chromatin.

### Quantitative RT-PCR (qRT-PCR)

Cell lysates were homogenized with the QiaShredder Column (Qiagen) and total cellular RNA was extracted using RNasy Mini kit (Qiagen) following the manufacturer’s instructions. Equal amounts of RNA (1 µg) were used for reverse transcription using the Primescript RT Reagent kit (Takara Bio) following the manufacturer’s instructions. cDNA was then diluted 1:20 and amplified in quantitative PCR which was monitored using Lightcycler 480 SYBR Green I Master (Roche Diagnostics, Belgium) in a LightCycler 480 Real-Time PCR System thermocycler (Roche Applied Science) and the results were analyzed using the ΔΔC_*T*_ method. The primers used were as follows: *GAPDH* 5′-AGCCACATCGCTCAGACAC-3′ (forward), 5′-GCCCAATACGACCAAAT CC-3′ (reverse); *SOCS3* 5′-CTTCGACTGCGTGCTAA-3′ (forward), 5′-GTAGGTGGCGAGGGG AAG-3′ (reverse); 2′5′*OAS* 5′-GACGGATGTTAGCCTGCTG-3′ (forward), 5′-TGGGGATTTGG TTTGGTG-3′ (reverse); *IFITM1* 5′-CACGCAGAAAACCACACTTC-3′ (forward), 5′-TGTTCCT CCTTGTGCATCTTC-3′ (reverse); *ISG54* 5′-TGGTGGCAGAAGAGGAAGAT-3′ (forward), 5′-G TAGGCTGCTCTCCAAGGAA-3′ (reverse). The fold change of mRNA expression was calculated by normalizing the relative amount to the internal control *GAPDH* and comparing with untreated cells.

### Immunofluorescence analysis and confocal microscopy

1.5 × 10^5^ HEK293T cells were seeded on coverslips previously coated with poly-L-lysine and grown in serum free medium for 16 h. After stimulation cells were washed once with PBS + Ca/Mg, fixated 15 minutes in 4% paraformaldehyde and washed three times with PBS + Ca/Mg. Cells were permeabilized 10 minutes at −20 °C with ice-cold methanol, washed once in PBS + Ca/Mg and incubated with blocking buffer (15.5 mM Na_2_HPO_4_; 4.5 mM NaH_2_PO_4_; 100 mM NaCl; 0.23% Triton X-100; 10% donkey serum) for 1 h and then 16 h at 4 °C with goat anti-Etag (Bethyl Laboratories) (1:2500) in blocking buffer. The day after, cells were washed 3 times with washing buffer (15.5 mM Na_2_HPO_4_; 4.5 mM NaH_2_PO_4_; 100 mM NaCl; 0.23% Triton X-100), followed by probing with donkey anti-goat secondary antibody (Alexa Fluor 488, Thermofisher Life Technologies) for 45 minutes (1:500) in blocking buffer at room temperature. Cells were then washed 3 times with washing buffer and once with PBS + Ca/Mg. 4,6-diamidino-2-phenylindole (DAPI) was used to stain the cell nuclei. A motorized inverted IX81 FluoView FV1000 laser scanning microscope (Olympus) was used to record high-resolution images.

### Molecular modeling

Phosphorylation of Y640 was modeled in YASARA structure^74^. A phosphate group was built on the tyrosine hydroxyl group of a STAT3 monomer (PDB code 1BG1, ref.^41^), followed by energy minimization using the AMBER99 force field. During four minimization steps, atoms within 0.25, 0.5 and 0.75 nm of the phosphate phosphorous atom were subsequently allowed to move, followed by minimization of all residues with any atom within 0.9 nm of this atom. Via the UCSF Chimera^75^ matchmaker protocol, the monomer STAT3 model with Y640 phosphorylation was superposed on the STAT3 dimer found in the crystal structure to test the compatibility of Y640 phosphorylation with the dimerization mode in the crystal structure.

### Electrophoretic Mobility-Shift Assay (EMSA)

HEK293T cells transiently transfected with the desired constructs were starved for 4 h in serum-free medium and were subsequently stimulated with 5 ng/ml IFNα2 or 15 ng/ml LIF for 15 min or were left untreated. Protein concentrations of the nuclear extracts were measured with the Bio-Rad protein assay. Double-stranded oligonucleotides based on the *cFOS* promoter (sense: 5′-GATCCGGGAGGGATTTACGGGAAATGCTG-3′; antisense: 5′-CTAGGCCCTCCCTAAATGCCCTTTACGAC-3′) and *SOCS3* promoter (sense: 5′-CGATTCCTGGAACTGCGCGG-3′; antisense: 5′-CAGCATTTCCGCGCAGTTCCAGGAA-3′) were labelled by filling in 5′ protruding ends with Klenow enzyme using (α-^32^P) dATP (3000 Ci/mmol; 10 mCi/ml; NEN) and purified using the Qiaquick PCR purification kit (Qiagen). Nuclear extracts (5 μg of protein) were incubated with approximately 10 fmol (20000 cpm) of probe in gel-shift incubation buffer (10 mM Hepes, pH 7.8, 1 mM EDTA, 5 mM MgCl_2_, 10% glycerol, 5 mM dithiothreitol, 2 mM Pefabloc® SC, 1 mg/ml BSA and 0.1 mg/ml poly(dI-dC)**·**(dI-dC)) for 10 min at room temperature (25 °C). The supershifting mouse anti-STAT3 antibody (Cell Signaling) was incubated with the nuclear extracts for 10 min at room temperature before addition of the radiolabeled probe. The protein–DNA complexes were separated on a 4.5% (w/v) polyacrylamide gel containing 7.5% glycerol in 0.5-fold TBE (Tris/borate/EDTA) at 20 V/cm for 90 min. Gels were fixed in water/methanol/ethanoic (acetic) acid (80:10:10, by vol.) for 30 min, dried and autoradiographed.

### Data Availability Statement

The datasets generated during and/or analysed during the current study are available from the corresponding author on reasonable request.

## Electronic supplementary material

Supplementary Data

## References

[1] Levy DE, Darnell JE (2002). Stats: transcriptional control and biological impact. Nat. Rev. Mol. Cell Biol..

[2] Mali SB (2015). Review of STAT3 (Signal Transducers and Activators of Transcription) in head and neck cancer. Oral Oncol..

[3] Jatiani SS, Baker SJ, Silverman LR, Reddy EP (2010). JAK/STAT Pathways in Cytokine Signaling and Myeloproliferative Disorders: Approaches for Targeted Therapies. Genes Cancer.

[4] Rane SG, Reddy EP (2002). JAKs, STATs and Src kinases in hematopoiesis. Oncogene.

[5] Constantinescu SN, Girardot M, Pecquet C (2008). Mining for JAK-STAT mutations in cancer. Trends Biochem. Sci..

[6] Casanova JL, Holland SM, Notarangelo LD (2012). Inborn Errors of Human JAKs and STATs. Immunity.

[7] Stark GR, Darnell JE (2012). The JAK-STAT Pathway at Twenty. Immunity.

[8] Darnell JE, Kerr IM, Stark GR (1994). Jak-STAT pathways and transcriptional activation in response to IFNs and other extracellular signaling proteins. Science.

[9] Yu H, Pardoll D, Jove R (2009). STATs in cancer inflammation and immunity: a leading role for STAT3. Nat. Rev. Cancer.

[10] Horvath CM, Darnell JE (1997). The state of the STATs: recent developments in the study of signal transduction to the nucleus. Curr. Opin. Cell Biol..

[11] Bowman T, Garcia R, Turkson J, Jove R (2000). STATs in oncogenesis. Oncogene.

[12] Haan C, Kreis S, Margue C, Behrmann I (2006). Jaks and cytokine receptors—An intimate relationship. Biochemical Pharmacology.

[13] Saharinen P, Silvennoinen O (2002). The pseudokinase domain is required for suppression of basal activity of Jak2 and Jak3 tyrosine kinases and for cytokine-inducible activation of signal transduction. J. Biol. Chem..

[14] Yeh TC, Dondi E, Uze G, Pellegrini S (2000). A dual role for the kinase-like domain of the tyrosine kinase Tyk2 in interferon-alpha signaling. Proc. Natl. Acad. Sci. USA.

[15] Yeh TC, Pellegrini S (1999). The Janus kinase family of protein tyrosine kinases and their role in signaling. Cell. Mol. Life Sci..

[16] Brown S, Zeidler MP (2008). Unphosphorylated STATs go nuclear. Curr. Opin. Genet. Dev..

[17] Reich NC (2013). STATs get their move on. Jak-Stat.

[18] Zhang X, Darnell JE (2001). Functional Importance of Stat3 Tetramerization in Activation of theα2-Macroglobulin Gene. J. Biol. Chem..

[19] Ndubuisi MI, Guo GG, Fried VA, Etlinger JD, Sehgal PB (1999). Cellular physiology of STAT3: Where’s the cytoplasmic monomer?. J. Biol. Chem..

[20] Aznar S (2001). Simultaneous tyrosine and serine phosphorylation of STAT3 transcription factor is involved in Rho A GTPase oncogenic transformation. Mol. Biol. Cell.

[21] Kim LC, Song L, Haura EB (2009). Src kinases as therapeutic targets for cancer. Nat Rev Clin Oncol.

[22] Akira S (1994). Molecular cloning of APRF, a novel IFN-stimulated gene factor 3 p91-related transcription factor involved in the gp130-mediated signaling pathway. Cell.

[23] Wen Z, Zhong Z, Darnell JE (1995). Maximal activation of transcription by Stat1 and Stat3 requires both tyrosine and serine phosphorylation. Cell.

[24] Wang R, Cherukuri P, Luo J (2005). Activation of Stat3 sequence-specific DNA binding and transcription by p300/CREB-binding protein-mediated acetylation. J. Biol. Chem..

[25] Yuan Z-L, Guan Y-J, Chatterjee D, Chin YE (2005). Stat3 dimerization regulated by reversible acetylation of a single lysine residue. Science.

[26] Ray S, Boldogh I, Brasier AR (2005). STAT3 NH2-terminal acetylation is activated by the hepatic acute-phase response and required for IL-6 induction of angiotensinogen. Gastroenterology.

[27] Yang J (2010). Reversible methylation of promoter-bound STAT3 by histone-modifying enzymes. Proc. Natl. Acad. Sci. USA.

[28] Stark GR, Wang Y, Lu T (2011). Lysine methylation of promoter-bound transcription factors and relevance to cancer. Cell Res..

[29] Ray S (2014). Inducible STAT3 NH2 terminal mono-ubiquitination promotes BRD4 complex formation to regulate apoptosis. Cell. Signal..

[30] Zhou Z (2016). SUMOylation and SENP3 regulate STAT3 activation in head and neck cancer. Oncogene.

[31] Pilati C (2011). Somatic mutations activating STAT3 in human inflammatory hepatocellular adenomas. J. Exp. Med..

[32] Koskela HLM (2012). Somatic *STAT3* Mutations in Large Granular Lymphocytic Leukemia. N. Engl. J. Med..

[33] Ohgami RS (2013). STAT3 mutations are frequent in CD30+ T-cell lymphomas and T-cell large granular lymphocytic leukemia. Leukemia.

[34] Ishida F (2014). STAT3 gene mutations and their association with pure red cell aplasia in large granular lymphocyte leukemia. Cancer Sci..

[35] Jerez A (2012). STAT3 mutations unify the pathogenesis of chronic lymphoproliferative disorders of NK cells and T-cell large granular lymphocyte leukemia. Blood.

[36] Wegenka UM, Buschmann J, Lütticken C, Heinrich PC, Horn F (1993). Acute-phase response factor, a nuclear factor binding to acute-phase response elements, is rapidly activated by interleukin-6 at the posttranslational level. Mol. Cell. Biol..

[37] Wegenka UM (1994). The interleukin-6-activated acute-phase response factor is antigenically and functionally related to members of the signal transducer and activator of transcription (STAT) family. Mol. Cell. Biol..

[38] Akira S (1994). Molecular cloning of APRF, a novel IFN-stimulated gene factor 3 p91-related transcription factor involved in the gp130-mediated signaling pathway. Cell.

[39] Zhong, Z., Wen, Z. & Darnell, J. Stat3: a STAT family member activated by tyrosine phosphorylation in response to epidermal growth factor and interleukin-6. *Science* (*80*−*)*. **264** (1994). 10.1126/science.8140422 8140422

[40] Krissinel E, Henrick K (2007). Inference of macromolecular assemblies from crystalline state. J. Mol. Biol..

[41] Becker S, Groner B, Müller CW (1998). Three-dimensional structure of the Stat3beta homodimer bound to DNA. Nature.

[42] Sasse J (1997). Mutational analysis of acute-phase response factor/Stat3 activation and dimerization. Mol. Cell. Biol..

[43] Domoszlai T (2014). Consequences of the disease-related L78R mutation for dimerization and activity of STAT3. J. Cell Sci..

[44] Icardi L (2012). Opposed regulation of type I IFN-induced STAT3 and ISGF3 transcriptional activities by histone deacetylases (HDACS) 1 and 2. FASEB J..

[45] Eyckerman S (2001). Design and application of a cytokine-receptor-based interaction trap. Nat. Cell Biol..

[46] Platanias LC (2005). Mechanisms of type-I- and type-II-interferon-mediated signalling. Nat. Rev. Immunol..

[47] Hirano T, Ishihara K, Hibi M (2000). Roles of STAT3 in mediating the cell growth, differentiation and survival signals relayed through the IL-6 family of cytokine receptors. Oncogene.

[48] Wu YY, Bradshaw RA (2000). Activation of the Stat3 Signaling Pathway Is Required for Differentiation by Interleukin-6 in PC12-E2 Cells. J. Biol. Chem..

[49] Chapman, R. S. *et al*. In *Biology of the Mammary Gland* 129–138 10.1007/0-306-46832-8_16 (Kluwer Academic Publishers, 2002).

[50] Flørenes VA (1999). Interleukin-6 dependent induction of the cyclin dependent kinase inhibitor p21WAF1/CIP1 is lost during progression of human malignant melanoma. Oncogene.

[51] Jang Y-N, Baik EJ (2013). JAK-STAT pathway and myogenic differentiation. JAK-STAT.

[52] O’Farrell, A.-M. *et al*. Stat3-Dependent Induction of p19INK4D by IL-10 Contributes to Inhibition of Macrophage Proliferation. *J. Immunol*. **164** (2000). 10.4049/jimmunol.164.9.4607 10779764

[53] Matsuda T (1999). STAT3 activation is sufficient to maintain an undifferentiated state of mouse embryonic stem cells. EMBO J..

[54] Bromberg JF (1999). Stat3 as an oncogene. Cell.

[55] Geng Y, Yu D, Blatt LM, Taylor MW (1995). Tumor suppressor activity of the human consensus type I interferon gene. Cytokines Mol. Ther..

[56] Icardi L (2012). The Sin3a repressor complex is a master regulator of STAT transcriptional activity. Proc. Natl. Acad. Sci..

[57] Nie Y (2009). STAT3 inhibition of gluconeogenesis is downregulated by SirT1. Nat. Cell Biol..

[58] Waitkus MS (2014). Signal integration and gene induction by a functionally distinct STAT3 phosphoform. Mol. Cell. Biol..

[59] Guschin D (1995). A major role for the protein tyrosine kinase JAK1 in the JAK/STAT signal transduction pathway in response to interleukin-6. Embo J..

[60] Stahl N (1994). Association and activation of Jak-Tyk kinases by CNTF-LIF-OSM-IL-6 beta receptor components. Science.

[61] Radtke S (2002). Novel Role of Janus Kinase 1 in the Regulation of Oncostatin M Receptor Surface Expression. J. Biol. Chem..

[62] Chung BM (2006). Jak2 and Tyk2 are necessary for lineage-specific differentiation, but not for the maintenance of self-renewal of mouse embryonic stem cells. Biochem. Biophys. Res. Commun..

[63] Karaghiosoff M (2000). Partial impairment of cytokine responses in Tyk2-deficient mice. Immunity.

[64] Sohn SJ (2013). A restricted role for TYK2 catalytic activity in human cytokine responses revealed by novel TYK2-selective inhibitors. J. Immunol..

[65] Crescenzo R (2015). Convergent mutations and kinase fusions lead to oncogenic STAT3 activation in anaplastic large cell lymphoma. Cancer Cell.

[66] Scarzello AJ (2007). A Mutation in the SH2 domain of STAT2 prolongs tyrosine phosphorylation of STAT1 and promotes type I IFN-induced apoptosis. Mol. Biol. Cell.

[67] Haan S (2000). Cytoplasmic STAT proteins associate prior to activation. Biochem. J..

[68] Kretzschmar AK, Dinger MC, Henze C, Brocke-Heidrich K, Horn F (2004). Analysis of Stat3 (signal transducer and activator of transcription 3) dimerization by fluorescence resonance energy transfer in living cells. Biochem. J..

[69] Domoszlai T (2014). Consequences of the disease-related L78R mutation for dimerization and activity of STAT3. J. Cell Sci..

[70] Yang CH, Murti A, Pfeffer LM (1998). STAT3 complements defects in an interferon-resistant cell line: evidence for an essential role for STAT3 in interferon signaling and biological activities. Proc. Natl. Acad. Sci. USA.

[71] Gamero AM (2006). Activation of Tyk2 and Stat3 is required for the apoptotic actions of interferon-beta in primary pro-B cells. J. Biol. Chem..

[72] Mahony, R. *et al*. A novel anti-viral role for STAT3 in IFN-α signalling responses. *Cell. Mol. Life Sci*. 1–10, 10.1007/s00018-016-2435-3 (2016). 10.1007/s00018-016-2435-3 PMC11107673 27988795

[73] Graham FL, van der Eb AJ (1973). A new technique for the assay of infectivity of human adenovirus 5 DNA. Virology.

[74] Krieger E (2009). Improving physical realism, stereochemistry, and side-chain accuracy in homology modeling: Four approaches that performed well in CASP8. Proteins: Structure, Function and Bioinformatics.

[75] Pettersen EF (2004). UCSF Chimera—A Visualization System for Exploratory Research and Analysis. J Comput Chem.

