# Supplementary material for: TYK2-induced phosphorylation of Y640 suppresses STAT3 transcriptional activity
Source: Sci Rep. 2017 Nov 21;7:15919. doi: 10.1038/s41598-017-15912-6 (PMC5698428; doi:10.1038/s41598-017-15912-6)
Supplement: Supplementary file 1 — Supplementary Data [file 41598_2017_15912_MOESM1_ESM.pdf]

TYK2-induced phosphorylation of Y640 suppresses STAT3 transcriptional activity

Mori Raffaele, Wauman Joris , Icardi Laura, Van der Heyden José , De Cauwer Lode, Peelman Frank, De Bosscher Karolien & Tavernier Jan.

Enhanced DNA binding of the STAT3 Y640F mutant.

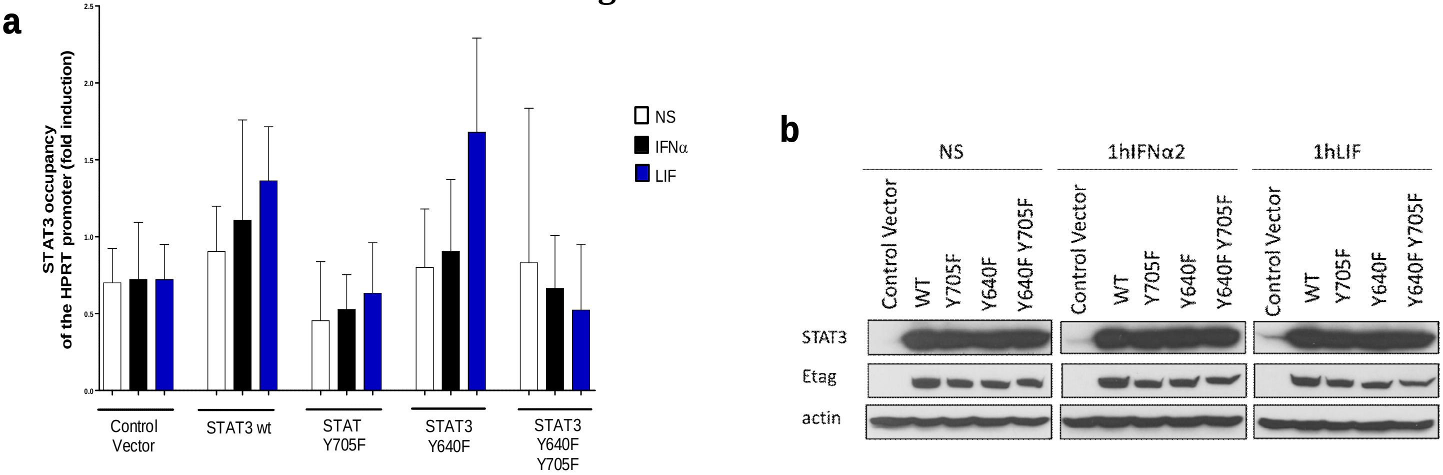

Supplementary Figure S1. ChIP assays were performed to examine the occupancy of STAT3 on the **a**) *HPRT* promoter. HEK293T cells were transiently transfected either with empty vector (Control Vector) or different Etag-STAT3 mutants: STAT3Y705F; STAT3 Y640F; STAT3 Y640F/Y705F. Cells were serum starved 4 hours and then left unstimulated (NS) or stimulated for 1 hour either with 10ng/ml IFNα2 or LIF. Immunoprecipitated DNA was used for qRT-PCR using specific primers for *HPRT* promoter. Graphs represent occupancy levels relative to irrelevant IgG immunoprecipitated DNA samples. All results are representative of 3 independent experiments. Error bars indicate SD. 1-way ANOVA with Bonferroni test. **b**) Total cells extracts were blotted and membranes were probed with anti-STAT3, anti-Etag and anti-β-actin antibodies. Full-length blots are presented in Supplementary Figures S14.

Enhanced DNA binding of the STAT3 Y640F mutant.

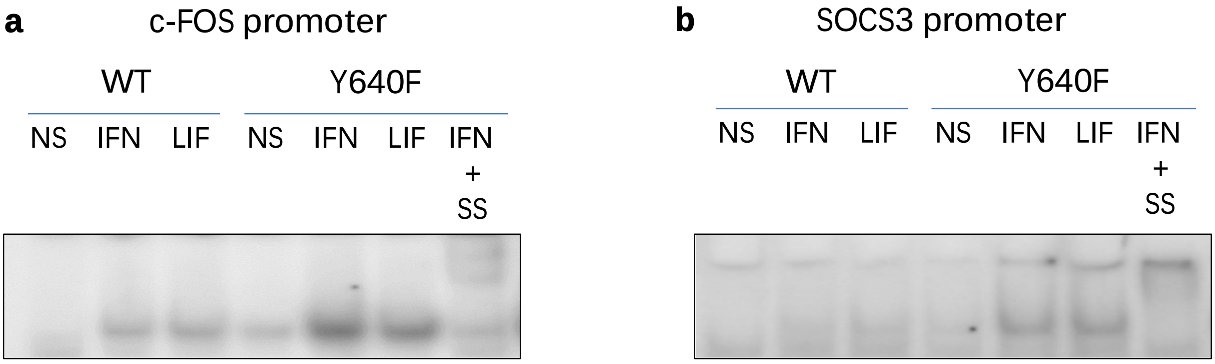

Supplementary Figure S2. EMSA showing the occupancy of STAT3 on the **a**) *c-FOS* and **b**) *SOCS3* promoter. HEK293T cells were transiently transfected with Etag-STAT3 or Etag-STAT3 Y640F. Cells were serum starved for 4 hours and then left unstimulated (NS) or stimulated for 15 minutes either with 5ng/ml IFNα2 or 15ng/ml LIF. Nuclear extracts were incubated with double-stranded labelled oligonucleotides based on the *c-FOS* promoter and *SOCS3* promoter. The supershift (SS) was obtained by incubating the nuclear extracts with mouse anti-STAT3 antibody before adding the labelled probe. The protein–DNA complexes were separated on polyacrylamide gel. Gels were fixed, dried and autoradiographed. Full-length gels are presented in Supplementary Figures S15.

Mutation of STAT3 at Y640 restores IFNαA-induced STAT3 transcriptional activity in murine NIH3T3 cells.

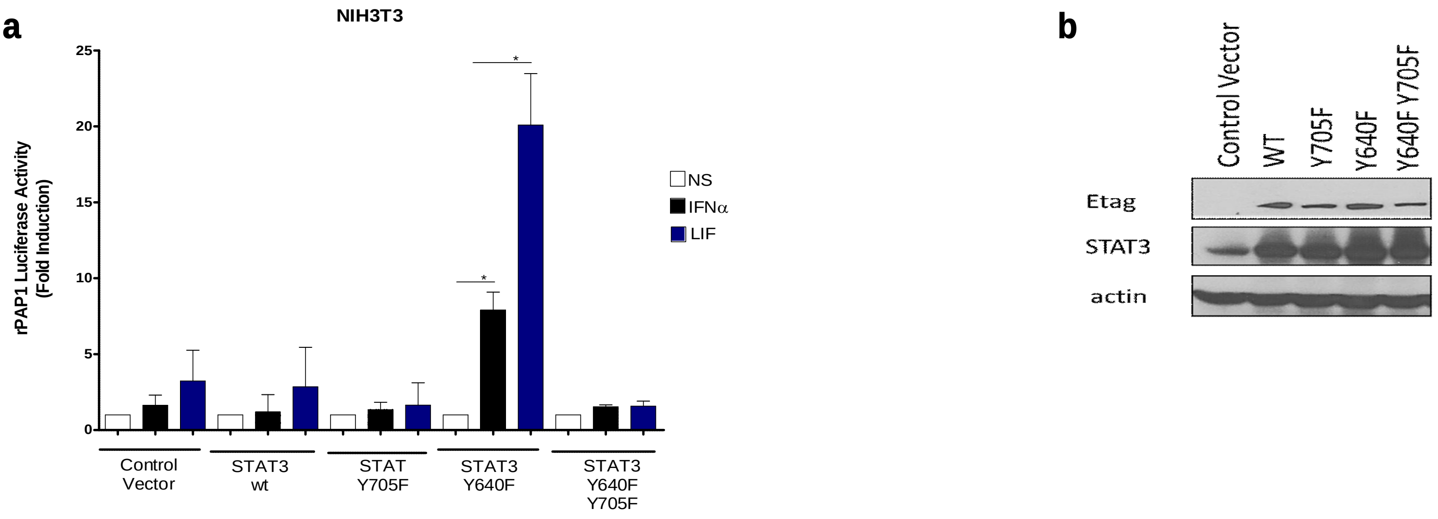

Supplementary Figure S3. Mutation of STAT3 at Y640 restores IFNα2-induced STAT3 transcriptional activity in murine NIH3T3 cells **a**) NIH3T3 cells were transiently transfected with the pXP2d2-rPAP1-luciferase and different pMet7-Etag-STAT3 Tyr mutants: STAT3Y705F; STAT3 Y640F; STAT3 Y640F/Y705F. Cells were serum starved 4 hours and then left unstimulated (NS) or stimulated for 24 hours either with 10ng/ml mIFNαA or hLIF. Luciferase readout is expressed as a ratio between stimulated and unstimulated values. All results are representative of 2 independent experiments. Error bars indicate SD. *P<0.05, **P<0.01, ***P<0.001; Student t-test. **b**) Cell lysates were blotted to test transfection efficiencies and the membranes probed with anti-STAT3, anti-Etag and anti-β-actin antibodies. Full-length blots are presented in Supplementary Figures S16.

Mutation of STAT3 at Y640 restores IFNαA-induced STAT3 transcriptional activity in murine N38 cells.

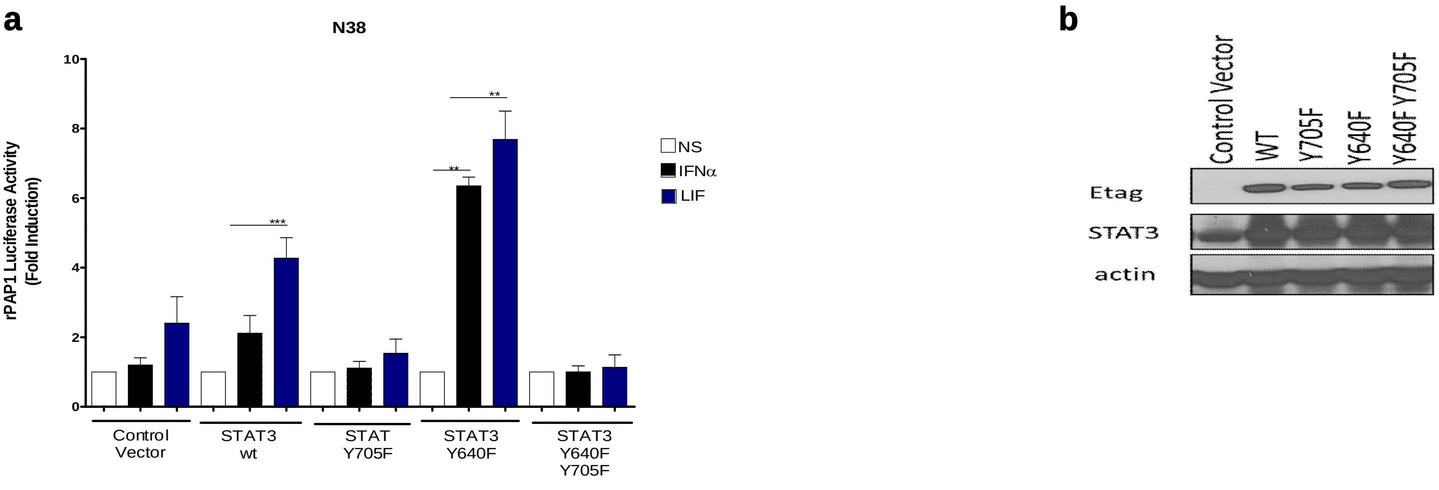

Supplementary Figure S4. Mutation of STAT3 at Y640 restores IFNα2-induced STAT3 transcriptional activity in murine N38 cells **a**) N38 cells were transiently transfected with the pXP2d2-rPAP1-luciferase and different pMet7-Etag-STAT3 Tyr mutants: STAT3Y705F; STAT3 Y640F; STAT3 Y640F/Y705F. Cells were serum starved 4 hours and then left unstimulated (NS) or stimulated for 24 hours either with 10ng/ml mIFNαA or hLIF. Luciferase readout is expressed as a ratio between stimulated and unstimulated values. All results are representative of 2 independent experiments. Error bars indicate SD. *P<0.05, **P<0.01, ***P<0.001; Student t-test. **b**) Cell lysates were blotted to test transfection efficiencies and the membranes probed with anti-STAT3, anti-Etag and anti-β-actin antibodies. Full-length blots are presented in Supplementary Figures S16.

Mutation of STAT3 at Y640 restores IFNα2-induced STAT3 transcriptional activity: mRNA levels of ISGF3 target gene *2'5'OAS.*

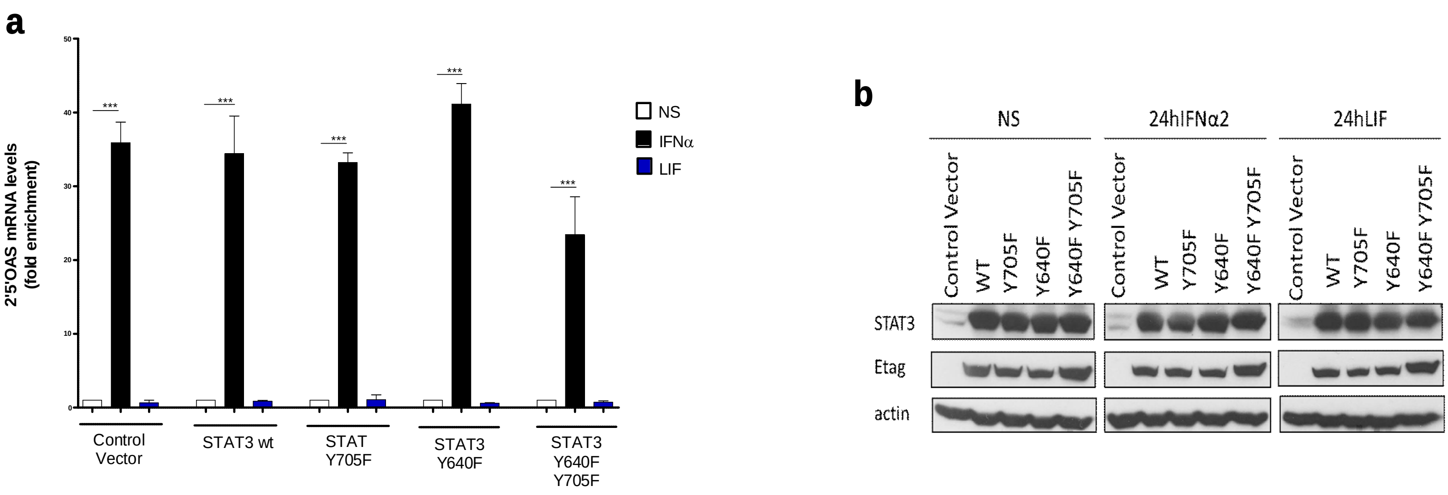

Supplementary Figure S5 . **a**) qRT-PCR analysis representing the relative mRNA levels of gene *2'5'OAS*. Hek293T cells were transiently transfected with empty vector pMet7 (control vector) or different pMet7-Etag-STAT3 Tyr mutants: STAT3Y705F; STAT3 Y640F; STAT3 Y640F/Y705F. Cells were serum starved 4 hours and then left unstimulated (NS) or stimulated for 24 hours either with 10ng/ml IFNα2 or LIF. Graphs represent the mRNA levels relative to the non-stimulated samples. All results are representative of 3 independent experiments. Error bars indicate SD from triplicates. ***P<0.001; 1-way ANOVA with Bonferroni test. **b**) Total cells extracts of HEK293T cells originating from RT-qPCR experiments of Fig.4e, Fig.4f and suppl. Data Fig. S5a were blotted and membranes were probed with anti-STAT3, anti-Etag and anti-β-actin antibodies. Full-length blots are presented in Supplementary Figures S17.

Supplementary Figure S6

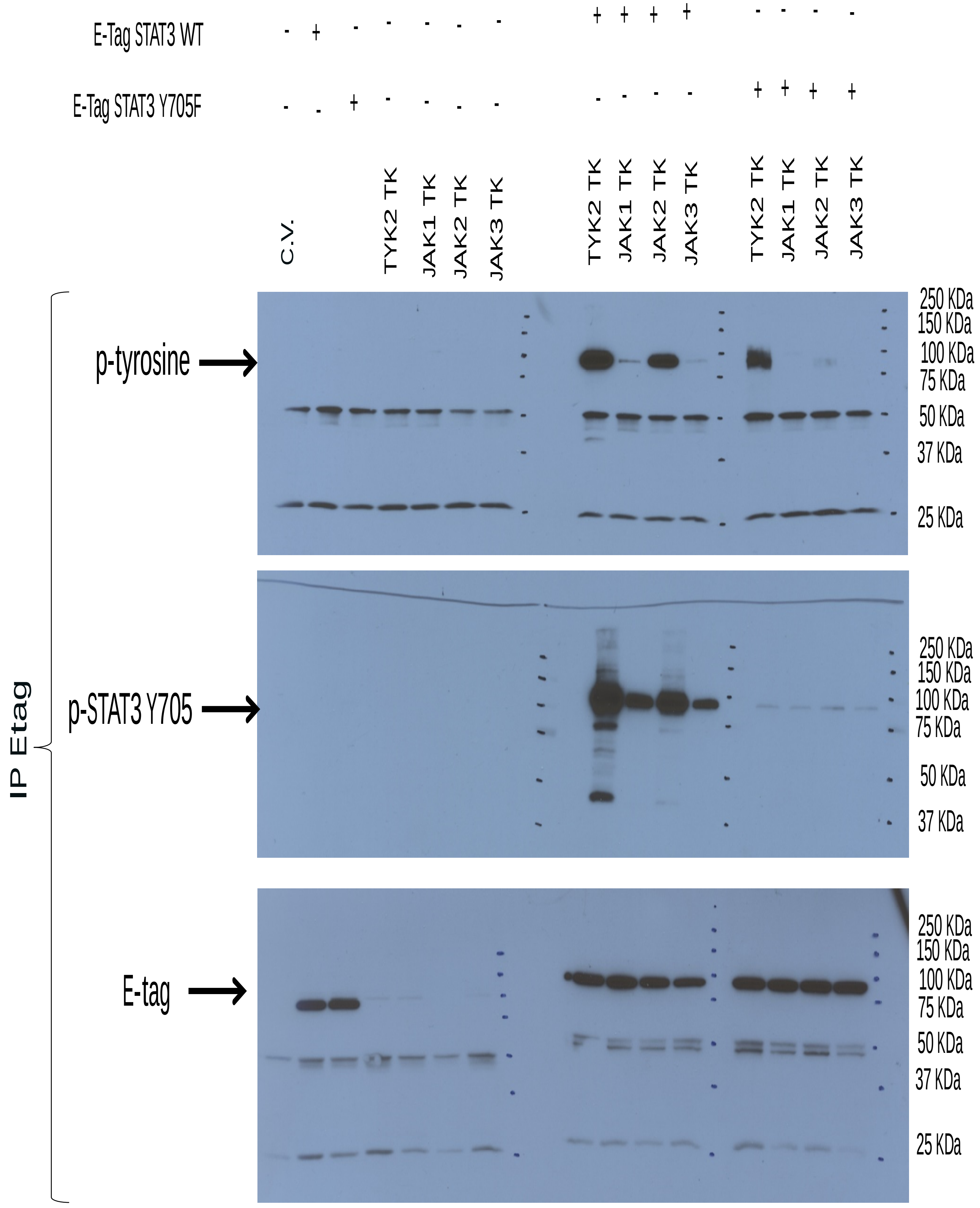

Supplementary Figure S6. Full-length Western blots of figure 1a. Immunoprecipitation of Etag-STAT3 with ProteinG Dynabeads and detection of phospho-tyrosine, phospho-STAT3 (pY705) and Etag. Information about probing procedure, primary and secondary antibodies are provided in the methods section.

Supplementary Figure S7

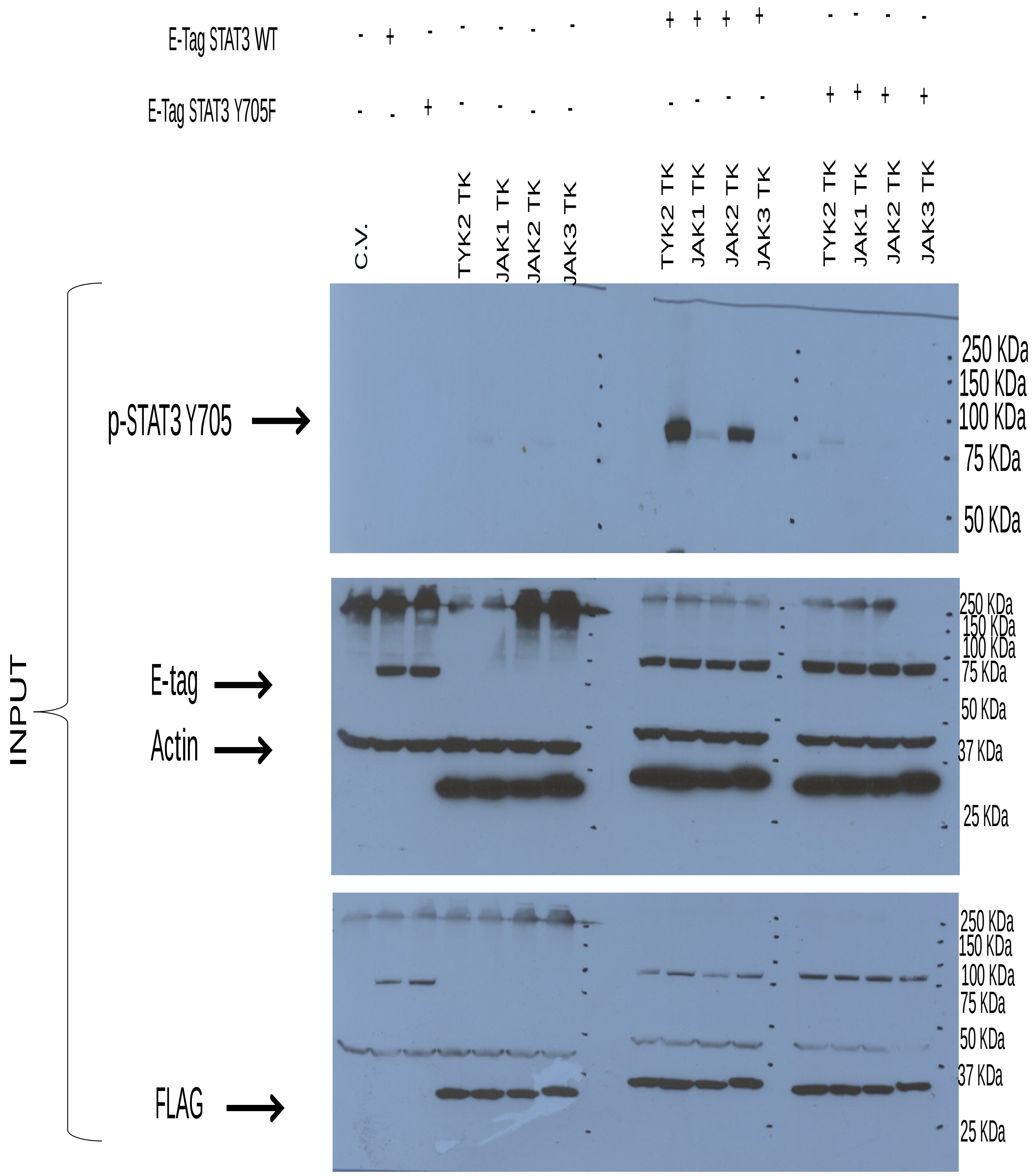

Supplementary Figure S7. Full-length Western blots of figure 1a. Total cell lysates were collected before immunoprecipitation, similar amounts of protein extracts were blotted for each condition, as quantified using a Bradford (Bio-Rad) assay and the membrane was probed with anti-phospho-STAT3 (pY705), anti-Etag, anti-β-actin and anti-FLAG antibodies. Information about probing procedure, primary and secondary antibodies are provided in the methods section.

Supplementary Figure S8

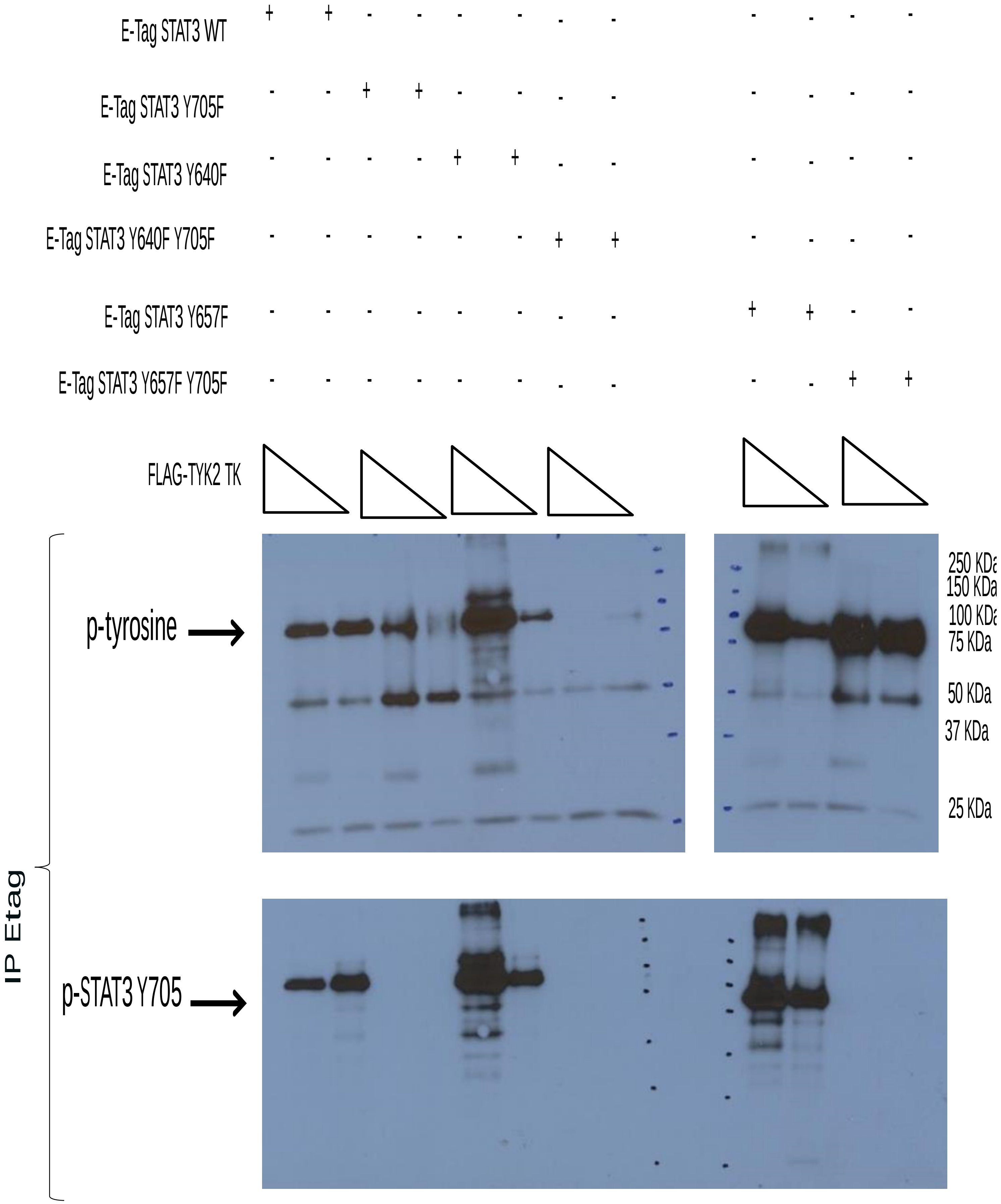

Supplementary Figure S8. Full-length Western blots of figure 1d. Immunoprecipitation of Etag-STAT3 with ProteinG Dynabeads and detection of phospho-tyrosine and phospho-STAT3 (pY705). Information about probing procedure, primary and secondary antibodies are provided in the methods section.

Supplementary Figure S9

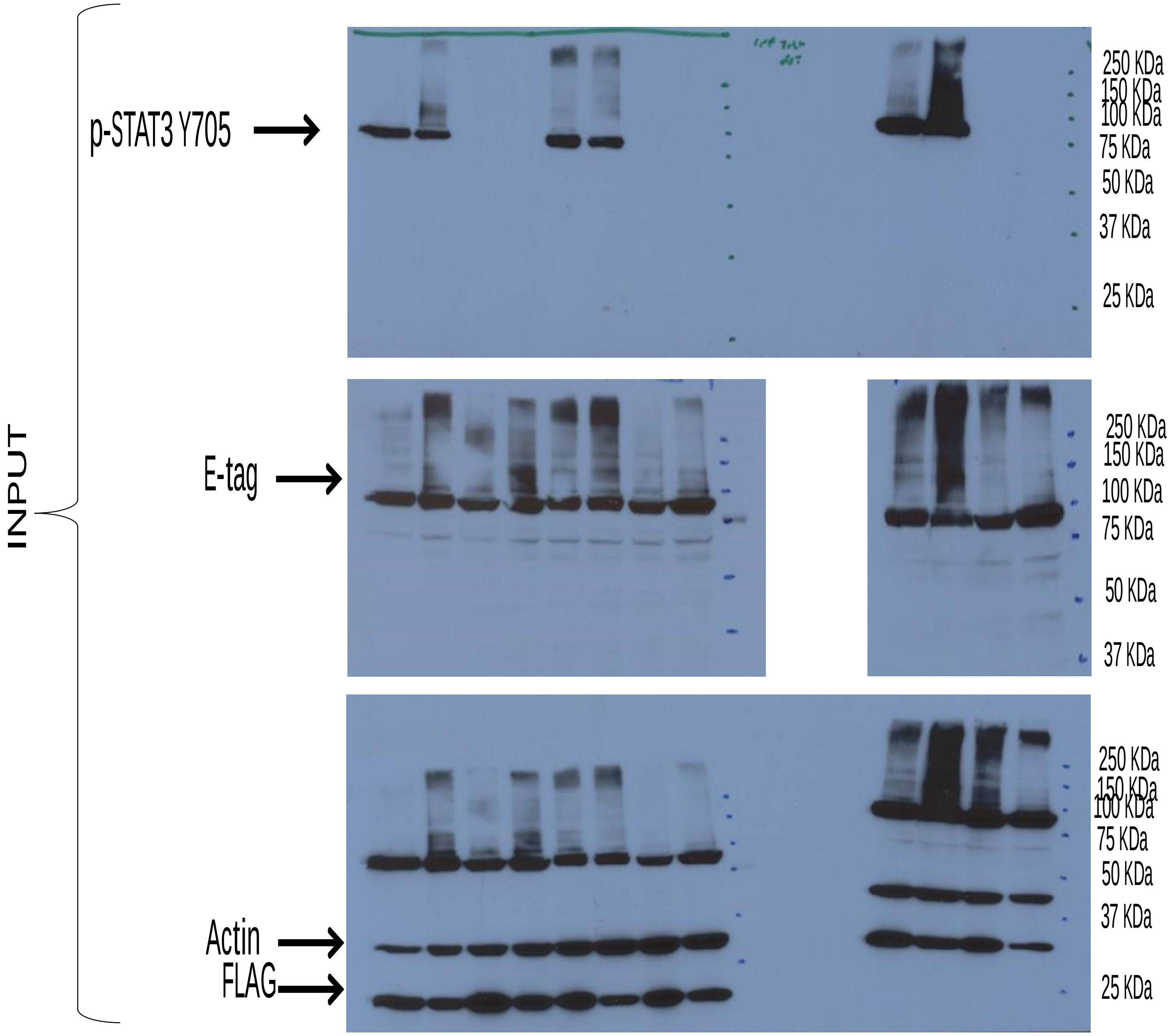

Supplementary Figure S9. Full-length Western blots of figure 1d. Total cell lysates were collected before immunoprecipitation, similar amounts of protein extracts were blotted for each condition, as quantified using a Bradford (Bio-Rad) assay and the membrane was probed with anti-phospho-STAT3 (pY705), anti-Etag, anti-β-actin and anti-FLAG antibodies. Information about probing procedure, primary and secondary antibodies are provided in the methods section.

Supplementary Figure S10

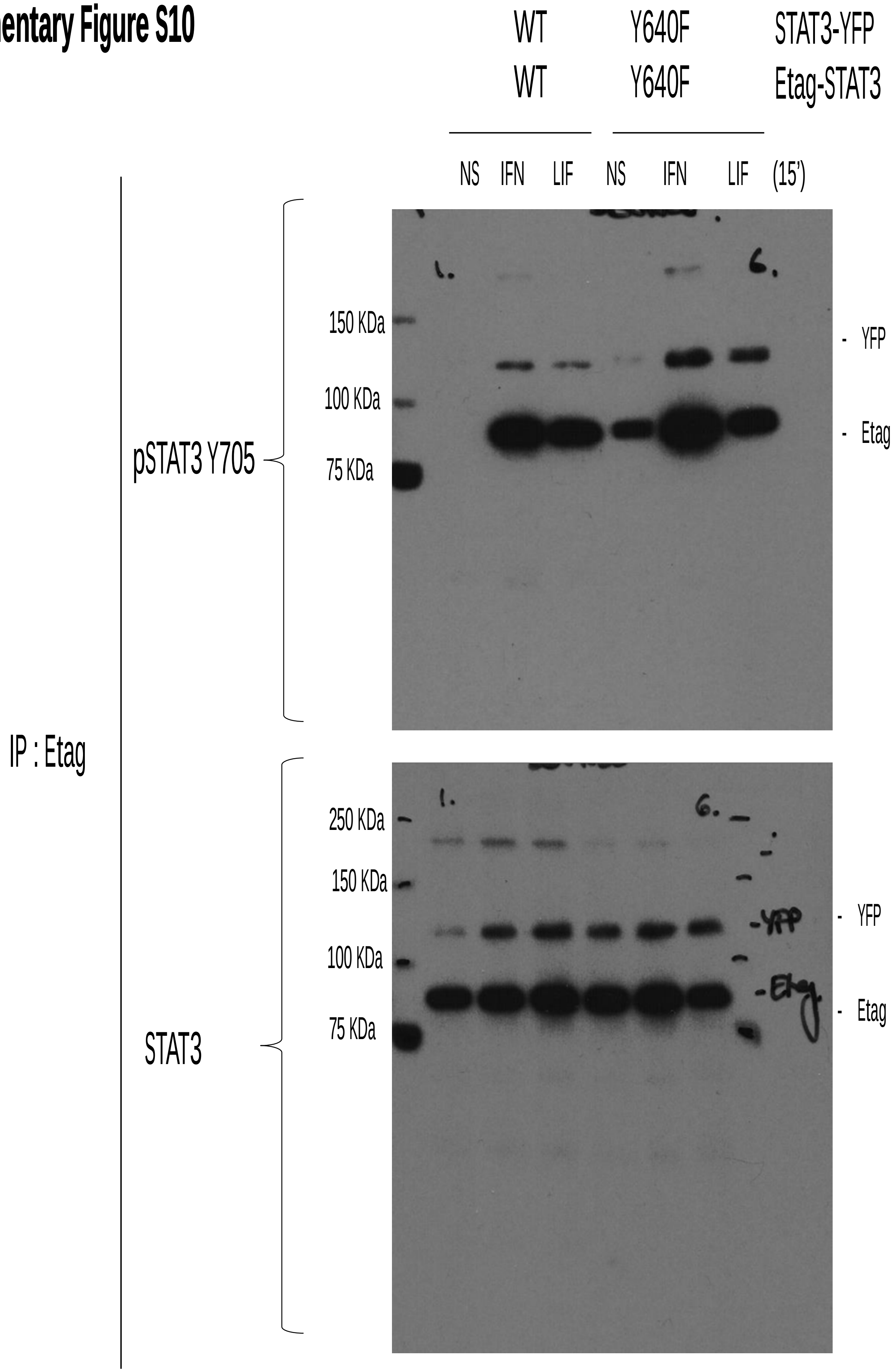

Supplementary Figure S10. Full-length Western blots of figure 2. Immunoprecipitation of Etag-STAT3 with ProteinG Dynabeads and detection of STAT3 and phospho-STAT3 (pY705). Information about probing procedure, primary and secondary antibodies are provided in the methods section.

Supplementary Figure S11

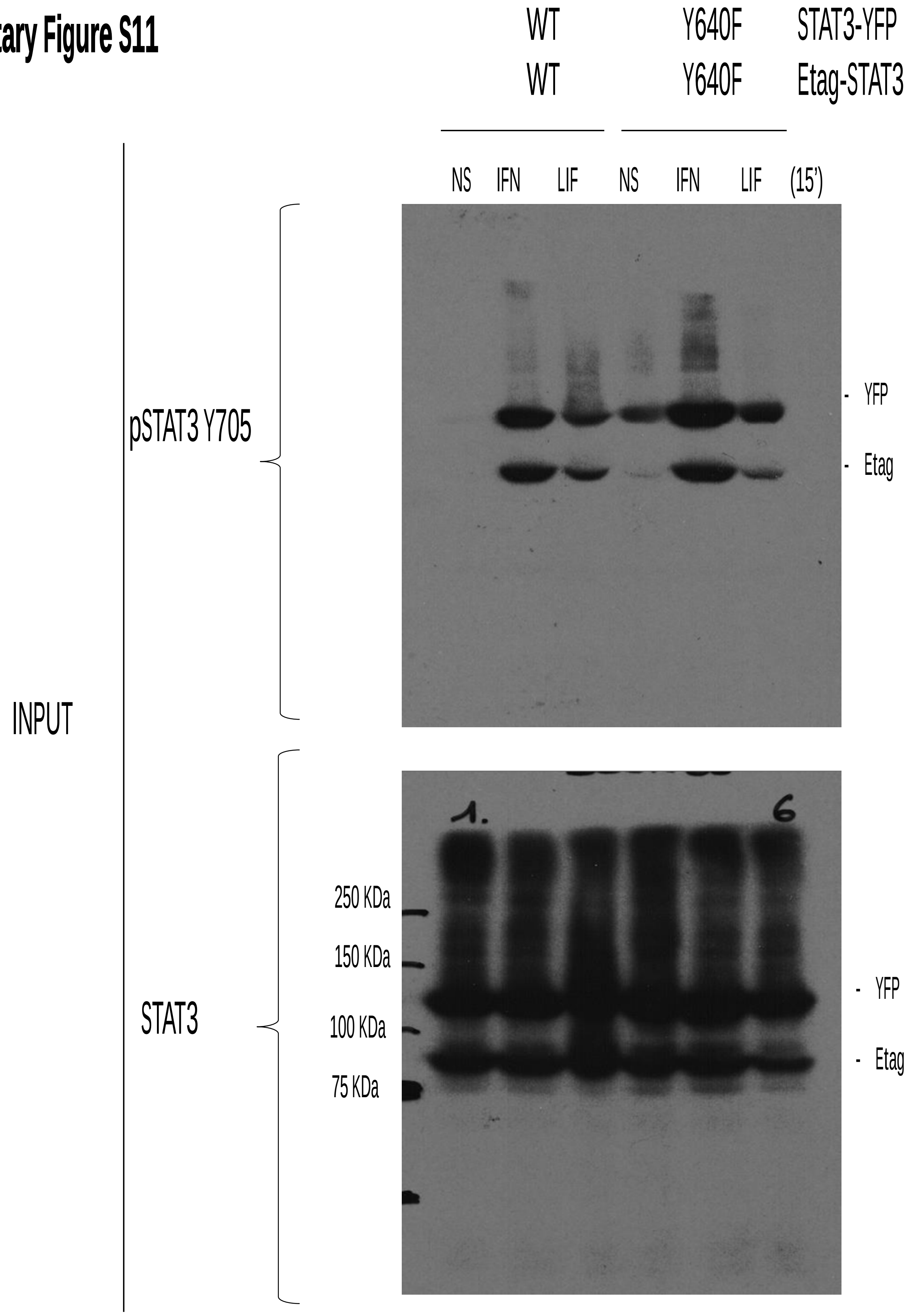

Supplementary Figure S11. Full-length Western blots of figure 2. Total cell lysates were collected before immunoprecipitation, similar amounts of protein extracts were blotted for each condition, as quantified using a Bradford (Bio-Rad) assay and the membrane was probed with anti-phospho-STAT3 (pY705), anti-Etag, anti-β-actin and anti-FLAG antibodies. Information about probing procedure, primary and secondary antibodies are provided in the methods section.

Supplementary Figure S12

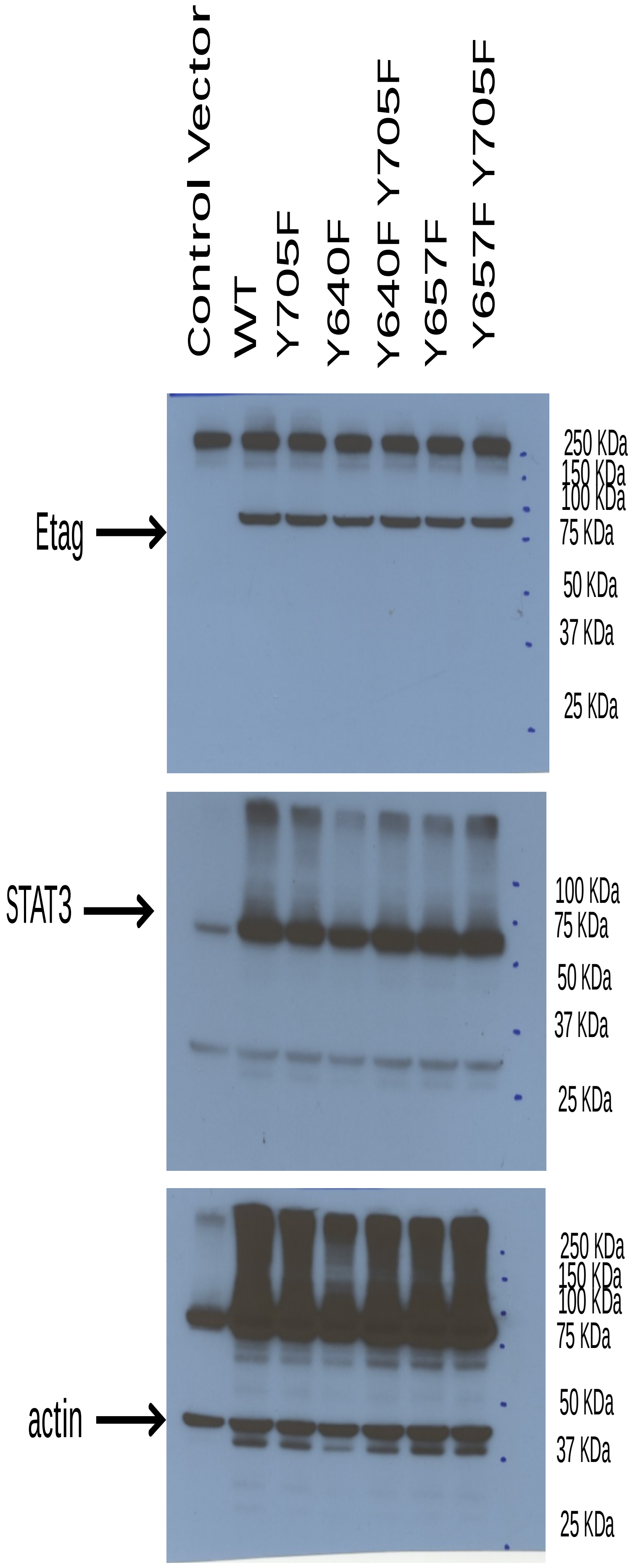

Supplementary Figure S12. Full-length Western blots of figure 4b. Total cells extracts were blotted to test transfection efficiencies, similar amounts of protein extracts were blotted for each condition, as quantified using a Bradford (Bio-Rad) assay and the membrane was probed with anti-STAT3, anti-Etag and anti-β-actin antibodies. Information about probing procedure, primary and secondary antibodies are provided in the methods section.

Supplementary Figure S13

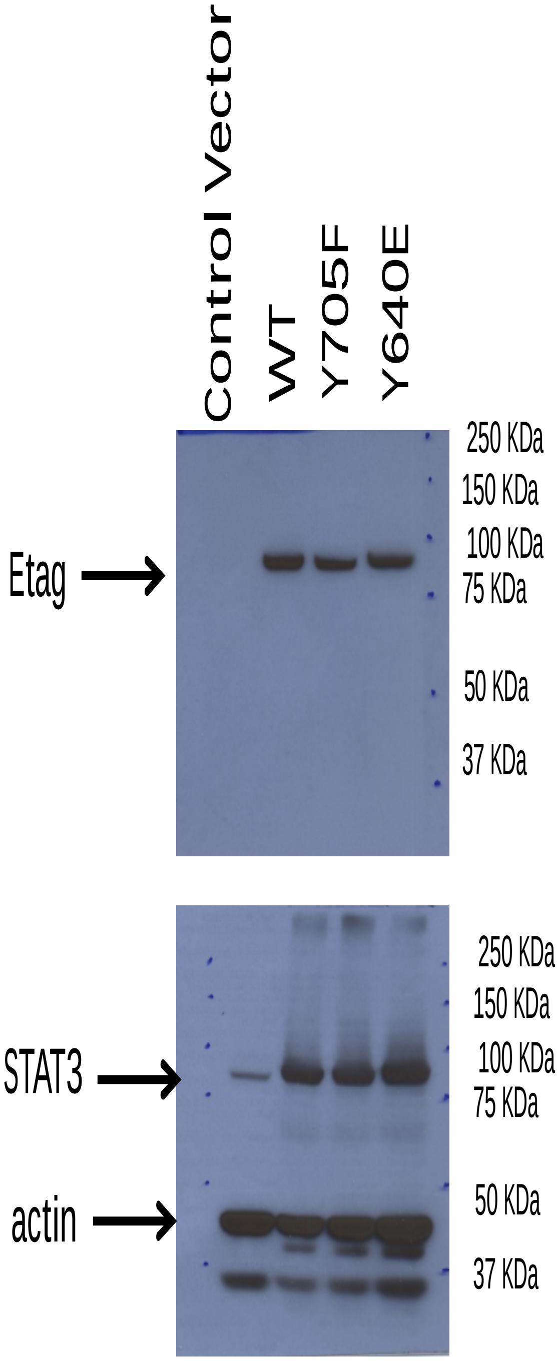

Supplementary Figure S13. Full-length Western blots of figure 4d. Total cells extracts were blotted to test transfection efficiencies, similar amounts of protein extracts were blotted for each condition, as quantified using a Bradford (Bio-Rad) assay and the membrane was probed with anti-STAT3, anti-Etag and anti-β-actin antibodies. Information about probing procedure, primary and secondary antibodies are provided in the methods section.

Supplementary Figure S14

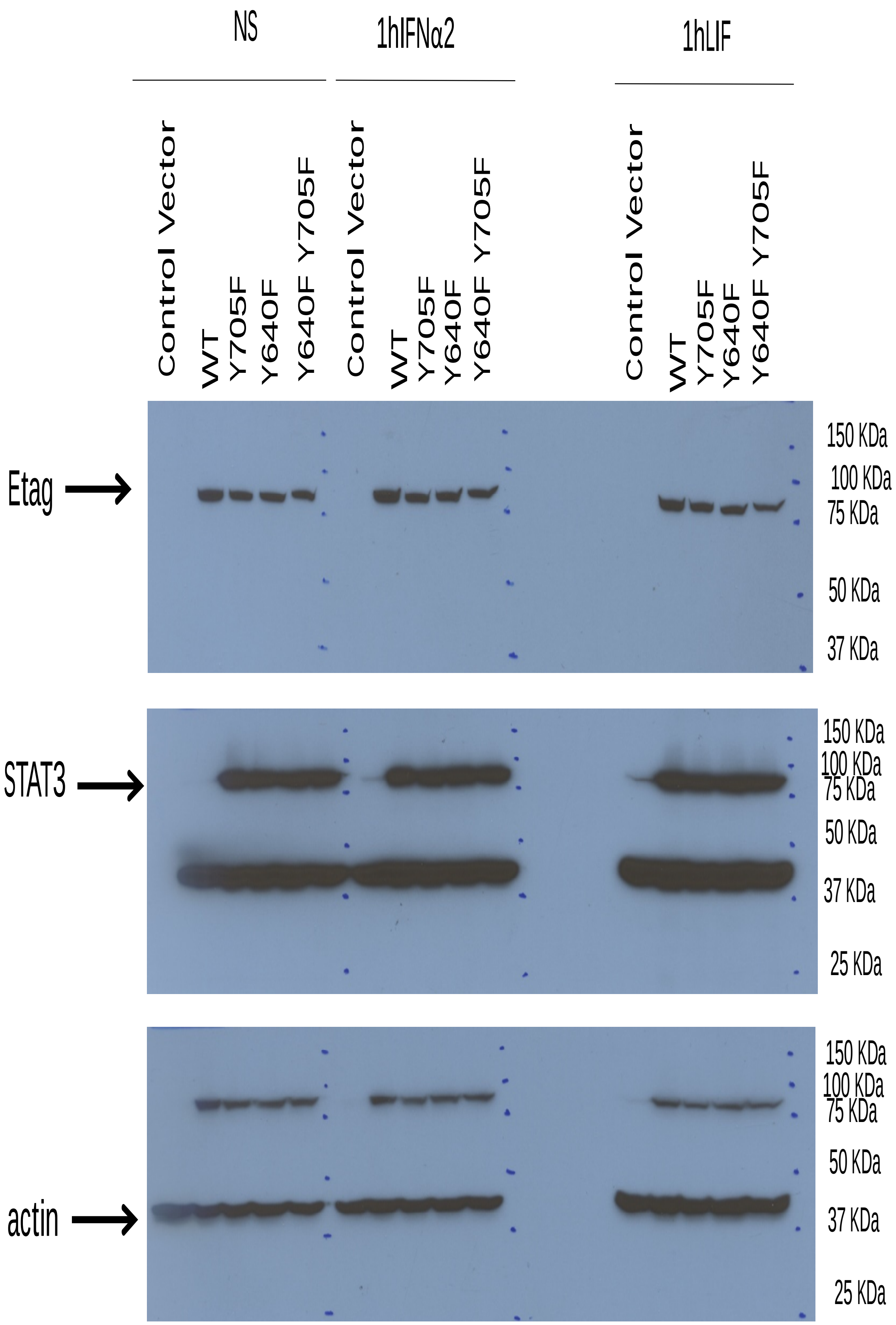

Supplementary Figure S14. Full-length Western blots of Supplementary Figure S1b. Total cells extracts were blotted to test transfection efficiencies, similar amounts of protein extracts were blotted for each condition, as quantified using a Bradford (Bio-Rad) assay and the membrane was probed with anti-STAT3, anti-Etag and anti-β-actin antibodies. Information about probing procedure, primary and secondary antibodies are provided in the methods section.

Supplementary Figure S15

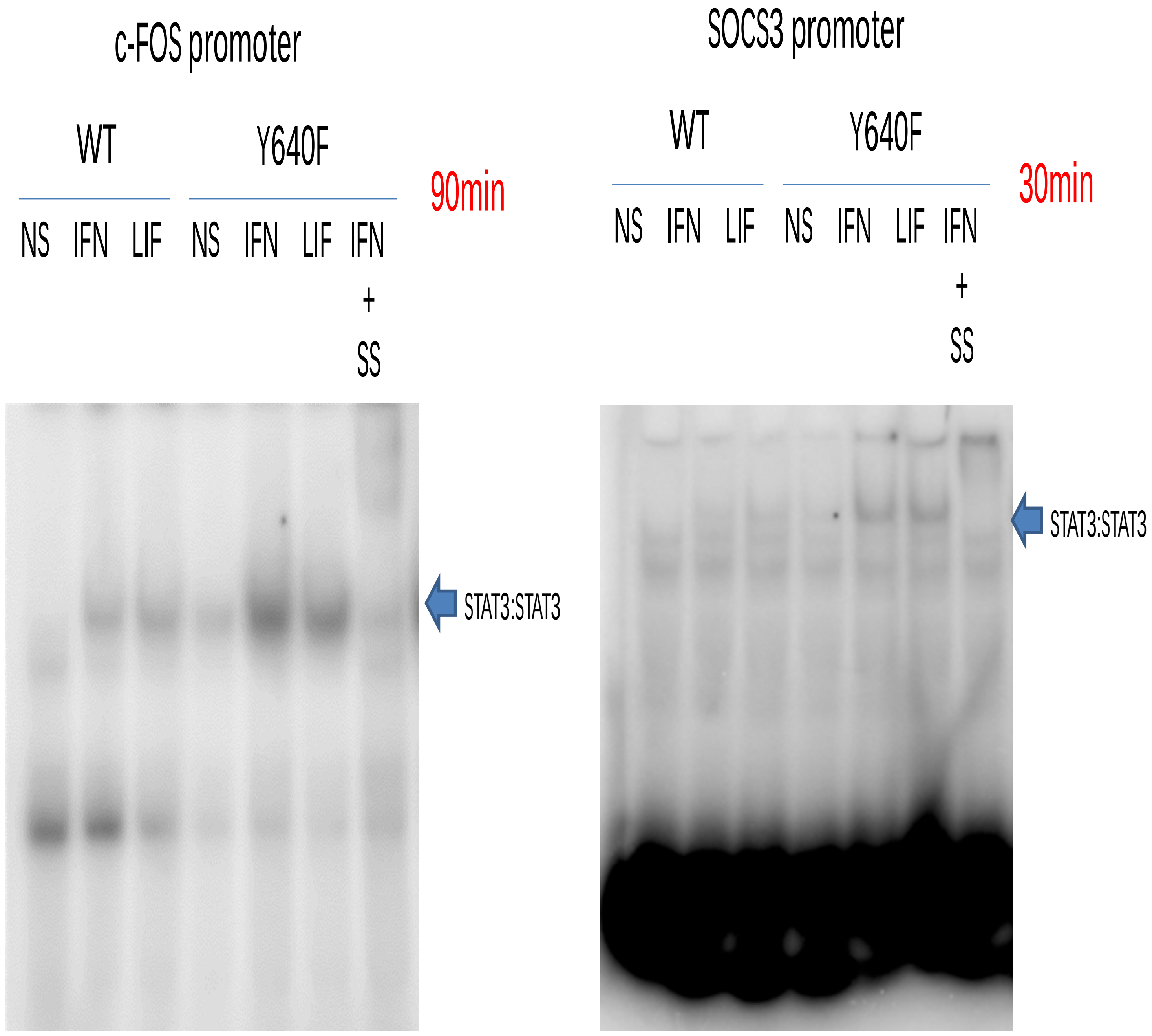

Supplementary Figure S15. Full-length gels of Supplementary Figure S2a-b. Nuclear extracts were incubated with double-stranded labelled oligonucleotides based on the c-FOS promoter and SOCS3 promoter. Bands corresponding to STAT3 dimers are indicated in the figure. Autoradiographic exposure time is indicated in red. Information about oligonuclotides, gel running conditions and antibodies are provided in the methods section.

Supplementary Figure S16

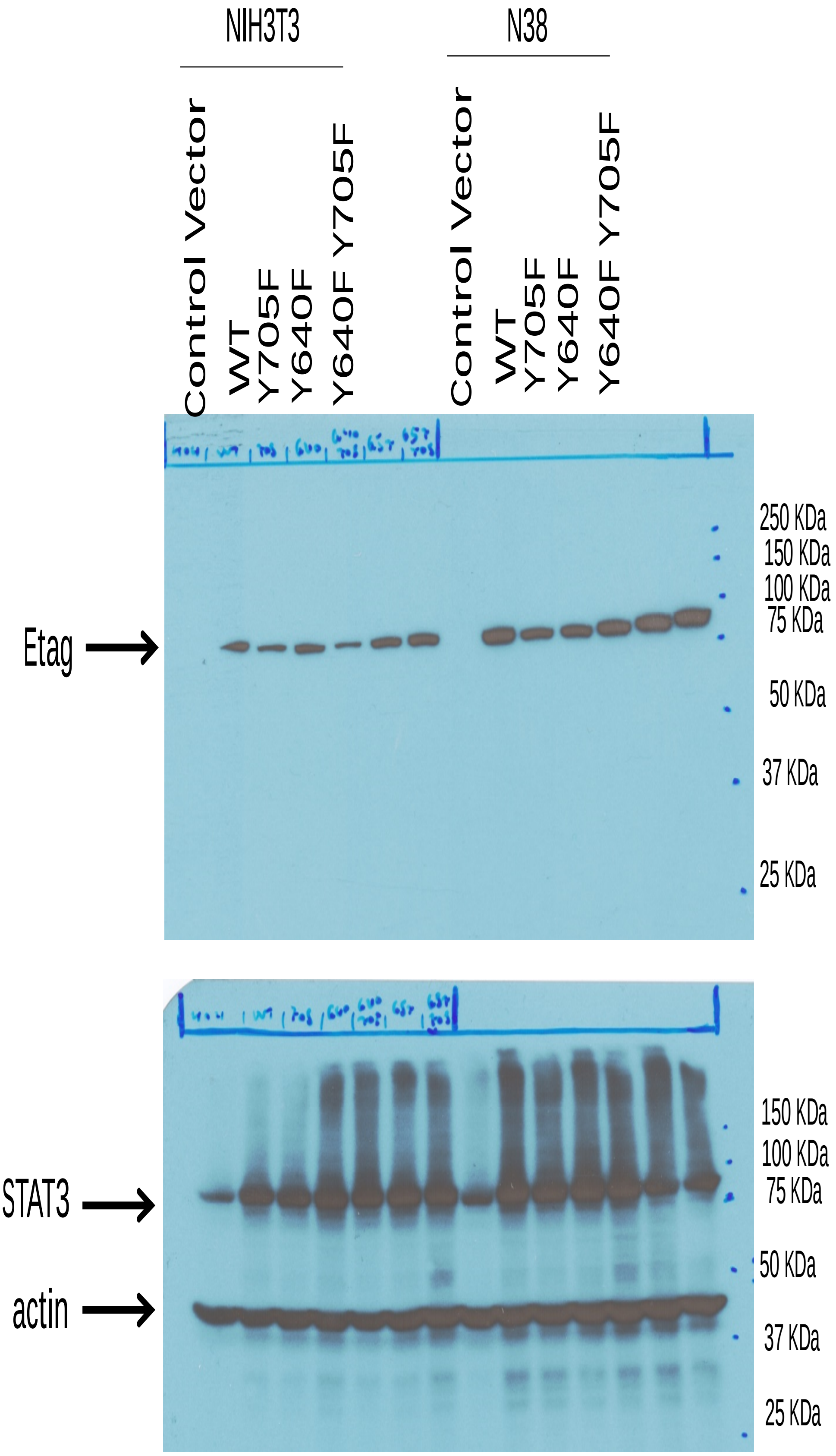

Supplementary Figure S16. Full-length Western blots of Supplementary Figure S3b-4b. Total cells extracts were blotted to test transfection efficiencies, similar amounts of protein extracts were blotted for each condition, as quantified using a Bradford (Bio-Rad) assay and the membrane was probed with anti-STAT3, anti-Etag and anti-β-actin antibodies. Information about probing procedure, primary and secondary antibodies are provided in the methods section.

Supplementary Figure S17

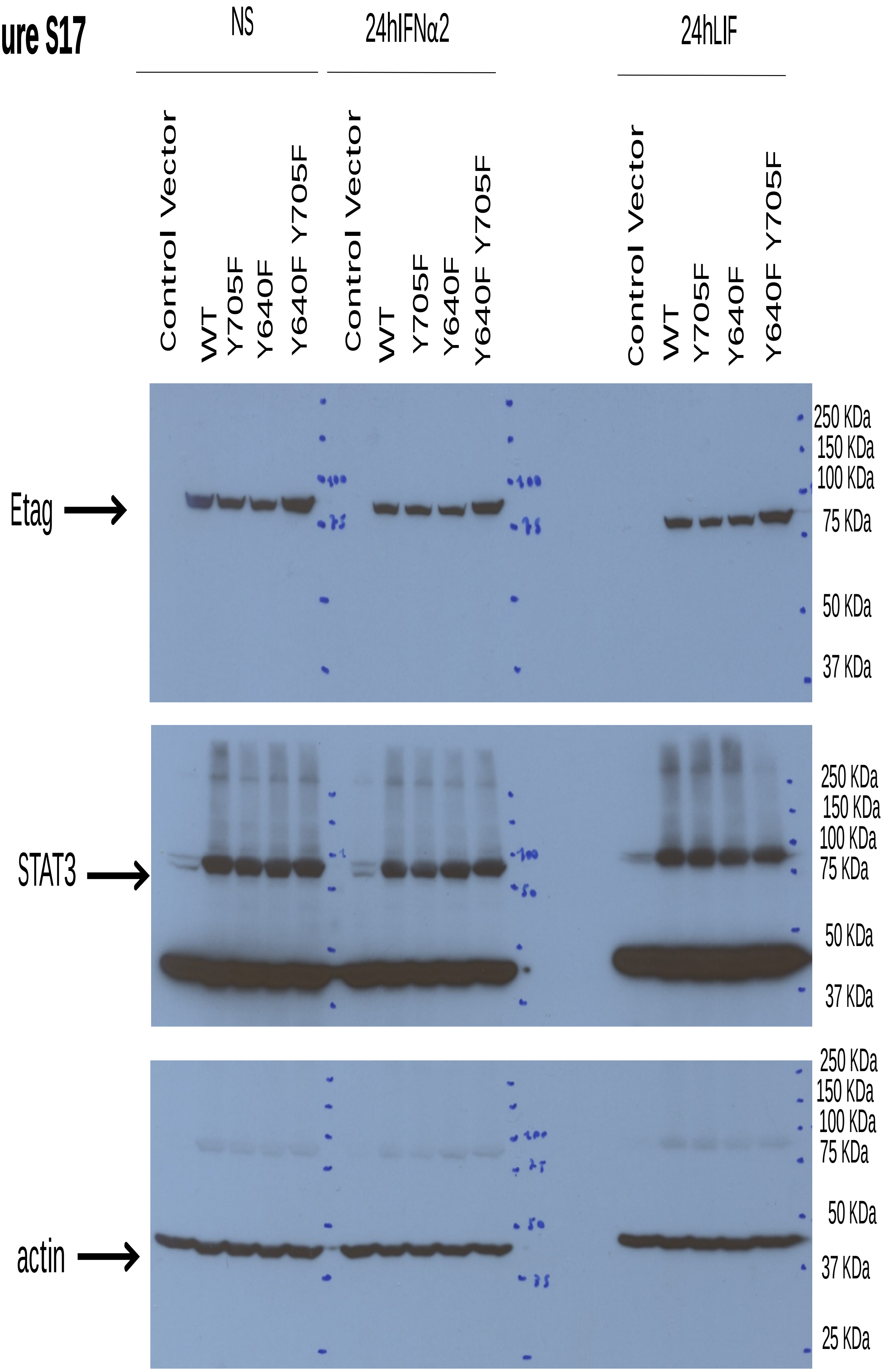

Supplementary Figure S17. Full-length Western blots of Supplementary Figure S5b. Total cells extracts were blotted to test transfection efficiencies, similar amounts of protein extracts were blotted for each condition, as quantified using a Bradford (Bio-Rad) assay and the membrane was probed with anti-STAT3, anti-Etag and anti-β-actin antibodies. Information about probing procedure, primary and secondary antibodies are provided in the methods section.
